# Single‐cell transcriptional atlas of human breast cancers and model systems

**DOI:** 10.1002/ctm2.70044

**Published:** 2024-10-17

**Authors:** Julia E. Altman, Amy L. Olex, Emily K. Zboril, Carson J. Walker, David C. Boyd, Rachel K. Myrick, Nicole S. Hairr, Jennifer E. Koblinski, Madhavi Puchalapalli, Bin Hu, Mikhail G. Dozmorov, X. Steven Chen, Yunshun Chen, Charles M. Perou, Brian D. Lehmann, Jane E. Visvader, J. Chuck Harrell

**Affiliations:** ^1^ Department of Human and Molecular Genetics Virginia Commonwealth University Richmond Virginia USA; ^2^ Department of Pathology Virginia Commonwealth University Richmond Virginia USA; ^3^ C. Kenneth and Diane Wright Center for Clinical and Translational Research Virginia Commonwealth University Richmond Virginia USA; ^4^ Department of Biochemistry Virginia Commonwealth University Richmond Virginia USA; ^5^ Massey Comprehensive Cancer Center Virginia Commonwealth University Richmond Virginia USA; ^6^ Department of Biostatistics Virginia Commonwealth University Richmond Virginia USA; ^7^ Department of Public Health Sciences University of Miami Miller School of Medicine Miami Florida USA; ^8^ Sylvester Comprehensive Cancer Center University of Miami Miller School of Medicine Miami Florida USA; ^9^ Walter and Eliza Hall Institute of Medical Research Melbourne Victoria Australia; ^10^ Department of Medical Biology University of Melbourne Parkville Victoria Australia; ^11^ Lineberger Comprehensive Cancer Center University of North Carolina Chapel Hill North Carolina USA; ^12^ Department of Medicine Vanderbilt University Medical Center Nashville Tennessee USA; ^13^ Center for Pharmaceutical Engineering Virginia Commonwealth University Richmond Virginia USA

**Keywords:** breast cancer, cellular heterogeneity, model limitations, preclinical research, single‐cell RNA sequencing, single‐cell transcriptomics, subtype‐specific insights, targetable pathways, therapeutic drug efficacy

## Abstract

**Background:**

Breast cancer's complex transcriptional landscape requires an improved understanding of cellular diversity to identify effective treatments. The study of genetic variations among breast cancer subtypes at single‐cell resolution has potential to deepen our insights into cancer progression.

**Methods:**

In this study, we amalgamate single‐cell RNA sequencing data from patient tumours and matched lymph metastasis, reduction mammoplasties, breast cancer patient‐derived xenografts (PDXs), PDX‐derived organoids (PDXOs), and cell lines resulting in a diverse dataset of 117 samples with 506 719 total cells. These samples encompass hormone receptor positive (HR+), human epidermal growth factor receptor 2 positive (HER2+), and triple‐negative breast cancer (TNBC) subtypes, including isogenic model pairs. Herein, we delineated similarities and distinctions across models and patient samples and explore therapeutic drug efficacy based on subtype proportions.

**Results:**

PDX models more closely resemble patient samples in terms of tumour heterogeneity and cell cycle characteristics when compared with TNBC cell lines. Acquired drug resistance was associated with an increase in basal‐like cell proportions within TNBC PDX tumours as defined with SCSubtype and TNBCtype cell typing predictors. All patient samples contained a mixture of subtypes; compared to primary tumours HR+ lymph node metastases had lower proportions of HER2‐Enriched cells. PDXOs exhibited differences in metabolic‐related transcripts compared to PDX tumours. Correlative analyses of cytotoxic drugs on PDX cells identified therapeutic efficacy was based on subtype proportion.

**Conclusions:**

We present a substantial multimodel dataset, a dynamic approach to cell‐wise sample annotation, and a comprehensive interrogation of models within systems of human breast cancer. This analysis and reference will facilitate informed decision‐making in preclinical research and therapeutic development through its elucidation of model limitations, subtype‐specific insights and novel targetable pathways.

**Key points:**

- Patient‐derived xenografts models more closely resemble patient samples in tumour heterogeneity and cell cycle characteristics when compared with cell lines.
- 3D organoid models exhibit differences in metabolic profiles compared to their in vivo counterparts.
- A valuable multimodel reference dataset that can be useful in elucidating model differences and novel targetable pathways.

## Graphical abstract

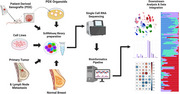

## 1 BACKGROUND

In 2023, breast cancer accounted for 31% of newly diagnosed cancer cases in women, making it the most commonly diagnosed cancer and the second leading cause of cancer‐related death among women in the United States.^1^, ^2^, ^3^ These malignancies exhibit extensive molecular heterogeneity^4^, ^5^, ^6^ and encompass diverse subtypes with distinct pathological responses.^4^, ^7^, ^8^ Recent advances in the genetic distinctions between various breast cancer subtypes have allowed for a more nuanced understanding of the molecular landscape underlying cancer formation and progression.^8^, ^9^ Breast cancers can be clinically categorised into groups based on estrogen receptor (ER) and progesterone receptor (PR) expression or human epidermal growth factor receptor 2 (HER2) overexpression, or by PAM50 gene signature scoring into four molecularly distinct subtypes: basal‐like, HER2‐enriched, luminal A, and luminal B. While subtype classification by immunohistochemistry staining (IHC) and PAM50 signature scoring of bulk tissues provide fundamentally important insights into expected patient outcome and appropriate treatment,^8^, ^9^, ^10^, ^11^ they provide limited insight into the functional implications of these subtypes at a cellular resolution.^10^, ^12^

Alongside increasing focus on subtype classifications, subtype‐specific pharmacologic targets are gaining attention. For this reason, HER2 amplified breast cancers and estrogen‐driven malignancies are now being treated with significant advancements due to more targeted therapeutic options.^13^, ^14^ Triple negative breast cancers (TNBCs), however, remain difficult to treat due to their lack of identified drug targets and significant transcriptional heterogeneity as defined by different TNBC types [Basal‐like 1 (BL1), Basal‐like 2 (BL2), Luminal androgen receptor (LAR), and Mesenchymal (M) subtypes].^15^, ^16^ Furthermore, TNBCs are among the worst prognosis cancers with high rates of metastasis and low patient survival.^17^, ^18^, ^19^, ^20^ A deeper understanding of the molecular diversity within these cancers is crucial to identifying targeted therapeutic strategies with meaningful implications for patients.

Advancements in integrated single‐cell RNA sequencing (scRNA‐seq) technologies have allowed for a deeper understanding of the fundamental biology and expression landscapes of a variety of cell types and tumour environments. The field of breast cancer research has greatly benefited from these technologies, playing a key role in elucidating potential therapeutic targets and identifying the development and origin of cancer.^21^ Furthermore, scRNA‐seq can help shed light on mechanisms underlying drug response, resistance to therapy,^22^, ^23^, ^24^ and cancer relapse.^25^ Single‐cell subtyping methodologies, such as SCSubtype and TNBCtype, have emerged to reveal intrinsic subtype heterogeneity within cancers.^10^, ^15^, ^16^ However, the comparative strengths of various subtyping methodologies and their application to various model systems is not well studied.

With these technologies, this study aims to integrate scRNA‐seq data from diverse breast cancer model systems and patient samples to delineate similarities and distinctions across models, explore stratification of therapeutic drug efficacy based on subtype proportions within tumours, and provide a dynamic approach to cell‐wise sample annotation. By leveraging this comprehensive 117 sample dataset, with 6 sample types, 7 different applied treatments, and isogenic pairs of drug resistance/sensitive models, we aim to contribute a high molecular resolution transcriptional atlas comprising human breast cancer cells from a variety of models and demonstrate the strengths of our dataset combined with our dynamic subtyping strategy to stratify and predict therapeutic treatment response.

## 2 RESULTS

### 2.1 Mapping cellular diversity in models of human breast cancer via scRNA‐seq integration

To investigate the global variations in breast heterogeneity among different models, we examined scRNA‐seq data that included transcriptional profiles from normal breast tissue, preneoplastic BRCA1+/– tissue, primary tumour samples from three clinical subtypes (ER+, HER2+, and TNBC),^26^ patient derived xenografts (PDXs), PDX‐derived organoids (PDXOs), cell lines, and several matched primary tumour associated lymph nodes. Leveraging the data from 117 distinct tissue specimens we obtained a dataset of over 500 000 human cells after quality control steps including filtering out murine and dead cells.

**FIGURE 1:**
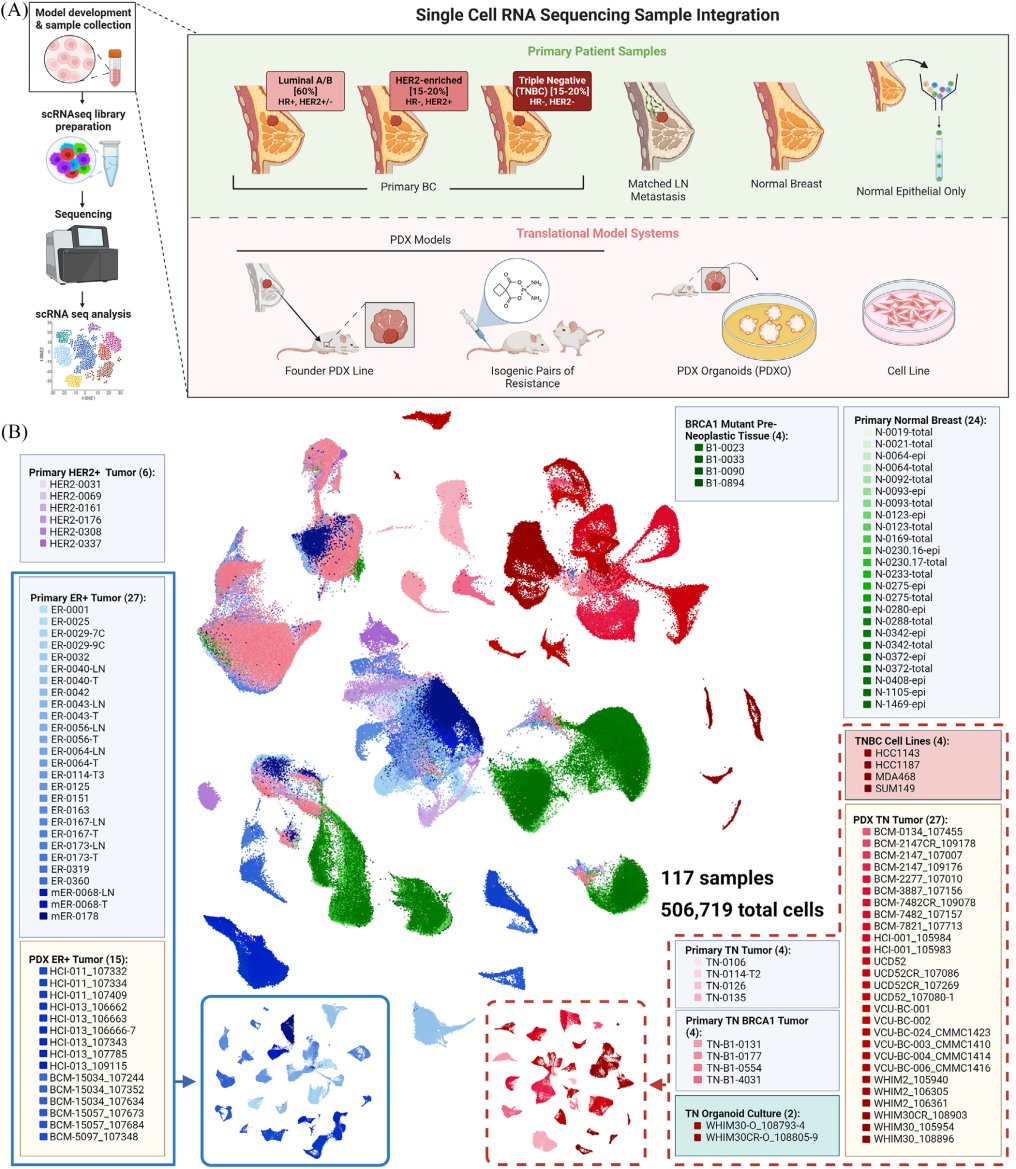
Data exploration schematic. (A) Diagram showing the sample processing pipeline beginning with sample collection and visually depicting the various model types used in this study. (B) An overview of the samples included within and different integration analysis performed, namely ER+ and TNBC typed sample mappings. Names of the samples are listed under each clustering diagram and the total number of samples shown in parentheses. UMAP visuals of ER+ (blue) and TNBC (red) malignant sample subsets outlined here. Of note, only malignant cell types were used when generating these subset UMAPs.

**FIGURE 2:**
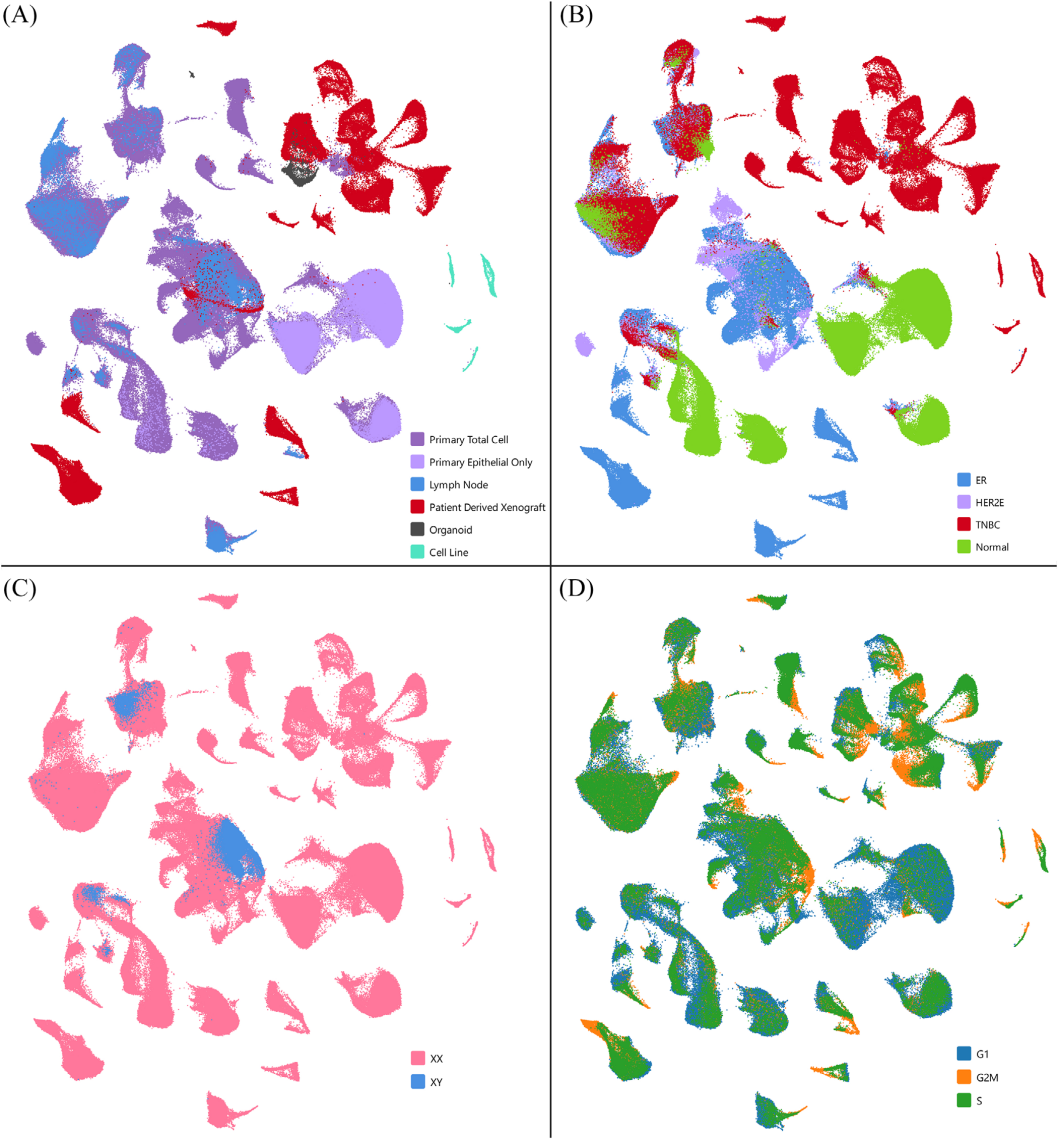
Data set visualisation by individual cells. UMAP visualisations, coloured by (A) tissue of origin, (B) clinical type, (C) X chromosome status, and (D) cell cycle phase.

Figure 1A illustrates the data collection approach and analysis pipeline, providing an overview of the types of samples and models involved in this study. Uniform Manifold Approximation and Projection (UMAP) visualisation of merged single‐cell RNA from all samples revealed distinct clustering patterns; clinically typed TNBC samples showed distinctly separate groupings, whereas primary HER2+ samples and primary ER+ samples were more closely transcriptionally related and seen to cluster together (Figures 1B, 2A and B, and S1). The positioning of PDX samples relative to primary samples of the same clinical type suggested an overall shared transcriptional program of the models and patient samples. However, we also note potential differences in transcriptional profiles between these model types, as PDX samples, while loosely grouped near primary samples of their shared clinical subtype, were seen to form distinct clusters from primary samples in many cases, especially within ER+ annotated samples (Figure 2A and B).

Reduced cellular heterogeneity was seen between models of breast cancer and primary/metastatic lesions, as demonstrated by UMAP dimensionality. Cell lines demonstrated the greatest reduction in heterogeneity with a comparatively more homogeneous cell population, as demonstrated by their tight clustering pattern, when compared to other sample types. Likewise, we observed increased heterogeneity in overall transcriptional profiles within primary samples compared to PDX or cell lines (Figures 1, 2, and S1). ER+ malignant samples from male (XY) origin were noted to cluster with other clinically typed ER+ malignant samples from female (XX) individuals (validated in 2 male patients), suggesting global gene expression patterns between these cells are not strongly influenced by sex‐related differences (Figures 1B and 2A–C).

To better define model and subtype‐specific differences, we examined proliferation and heterogeneity across diverse breast cancer models and patient samples within our dataset. Cell cycle phase was identified from gene expression for each cell. TNBC models tended to have a greater proportion of actively proliferating (G2M and S phase) cells compared to other clinical subtypes (Figures 2D and S2). This observation aligns with existing knowledge about the aggressive nature of TNBC, characterised by increased cellular proliferation.^18^, ^27^ Similar trends for proliferation were observed between model type within clinical subtypes. PDX, organoid, and cell line models displayed a trend towards greater proportions of cells in S and G2M phase compared to primary or metastatic lesions from patients, with cell lines having the highest proportion (Figure S2). Importantly, this information enriches our understanding of the intrinsic characteristics of breast cancer model systems, comparative to direct patient samplings.

### 2.2 Gene signature analysis reveals distinct immune, normal, and malignant cell clusters

**FIGURE 3:**
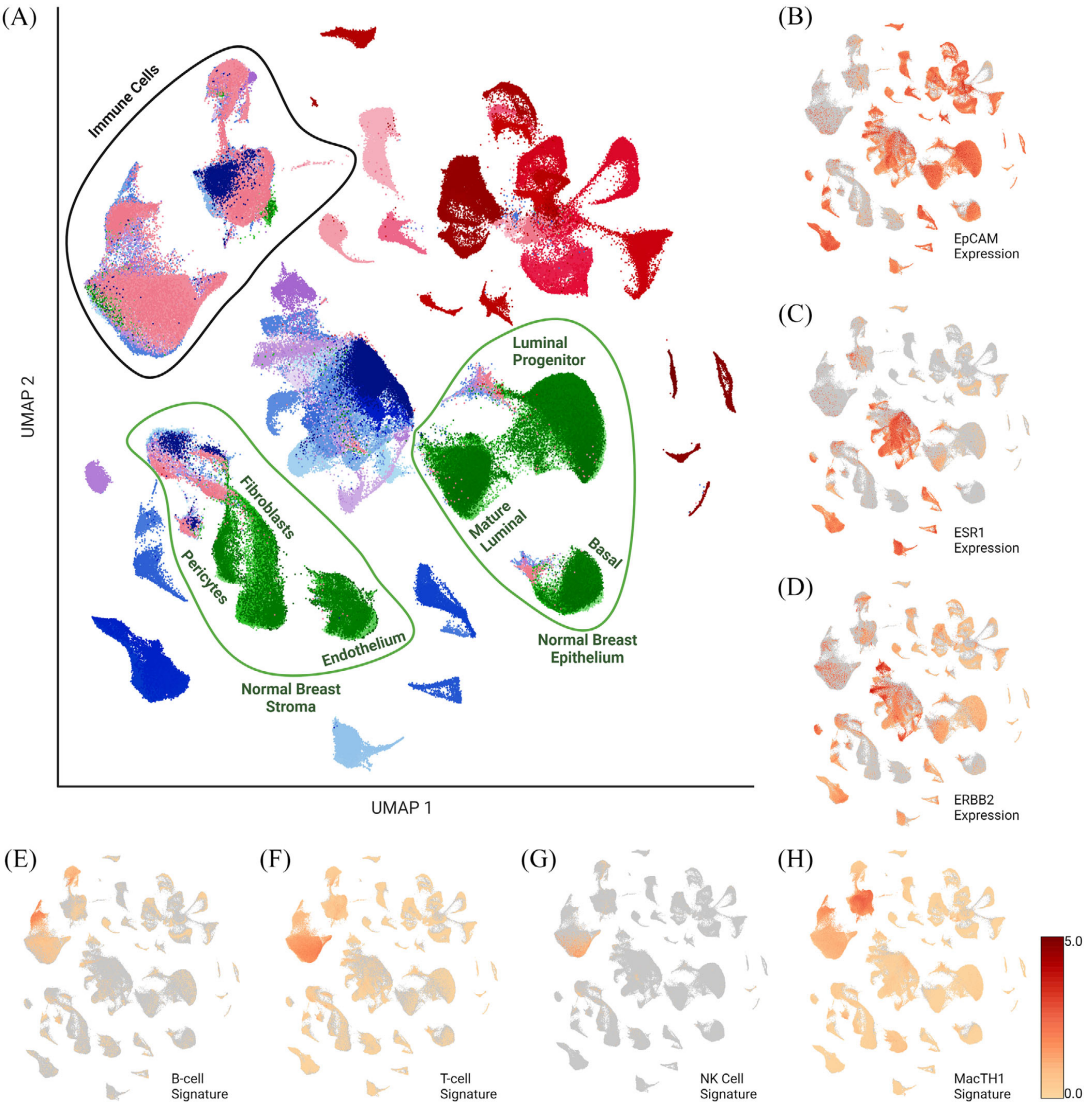
Identification and removal of non‐malignant cells. (A) Annotated clustering with identified cell types following gene signature analysis. UMAP heatmaps for key genes (B) EpCAM, (C) ESR1, (D) ERBB2 and immune signatures for (E) B cells, (F) T cells, (G) natural killer cells, and (H) combined signature for macrophages, monocyte, and myeloid‐derived cells. Visualisations using log normalised feature averages. Heatmap scale is log normalised average gene expression for each signature.

To better define cell clusters within this merged set, we utilised established gene signatures (Table S1) to define cell types (Figure 3A). We first identified epithelial cells both normal and malignant by the expression of epithelial cellular adhesion molecule (EpCAM) (Figure 3B).^28^ EpCAM^low^ clusters primarily contained non‐malignant cell types, with the exception of the metaplastic/claudin‐low^29^ PDX, BCM‐7482. HER2 (ERBB2) and ESR1 expression aligned with prior clinical subtyping of samples (Figure 3C and D).

Clusters formed by malignant cells were further validated using inferCNV method to infer copy number alterations from normal samples (Figure S3), providing an additional layer of confidence in discerning the malignant cell populations from the complex cellular milieu. InferCNV was not used as the primary method of malignant cell discernment as we noted that some primary ER+ and HER2 overexpressed tissue derived cells did not differ significantly from reference samples in copy number, despite clustering and gene expression aligning with malignant cell types. Normal cell clusters were inferred based on localisation of normal breast tissue cells (taken from reduction mammoplasties) merged into the dataset. Cells from malignant samples, which clustered with normal tissue cells, were assumed to be adjacent normal cells within the malignant tissues (Figure 3A). Leveraging previously defined immune signatures (Table S1) we were able to identify clusters containing cells with high expression for genes associated with several immune cell types (Figure 3E–H).

Following preliminary identification of normal cell clusters, we sought to further define these clusters. We used previously identified gene signatures for breast epithelium to identify basal (e.g., KRT5, ACTA2, MYLK, SNAI2), luminal progenitor [TNFRSF11A (RANK), KIT], and mature luminal cells (ESR1, PGR, FOXA1).^26^, ^30^ We were likewise able to identify clusters with low EpCAM expression which contained high expression of pericytes, fibroblasts, or endothelium associated cell signatures,^31^ annotated here as stromal cell populations within the normal cell clusters. These clusters have been annotated as shown in Figure 3A. The integrity of these normal cell clusters and conserved features from prior literature, provide further confidence of our normal cell cluster identification.

### 2.3 Differences in immune proportions between clinical subtypes of breast cancer

To examine transcriptional differences between tumour cells, we removed all non‐malignant epithelial, immune and stromal cells. Clusters of cells that could not be verified as malignant cell types and were in proximity to immune or normal cell clusters were systematically eliminated, ensuring a conservative approach in defining malignant cell clusters. This would allow for better comparison of the transcriptional profiles between models; as PDXs, PDXOs, and cell lines do not contain human immune or normal cell types.

**FIGURE 4:**
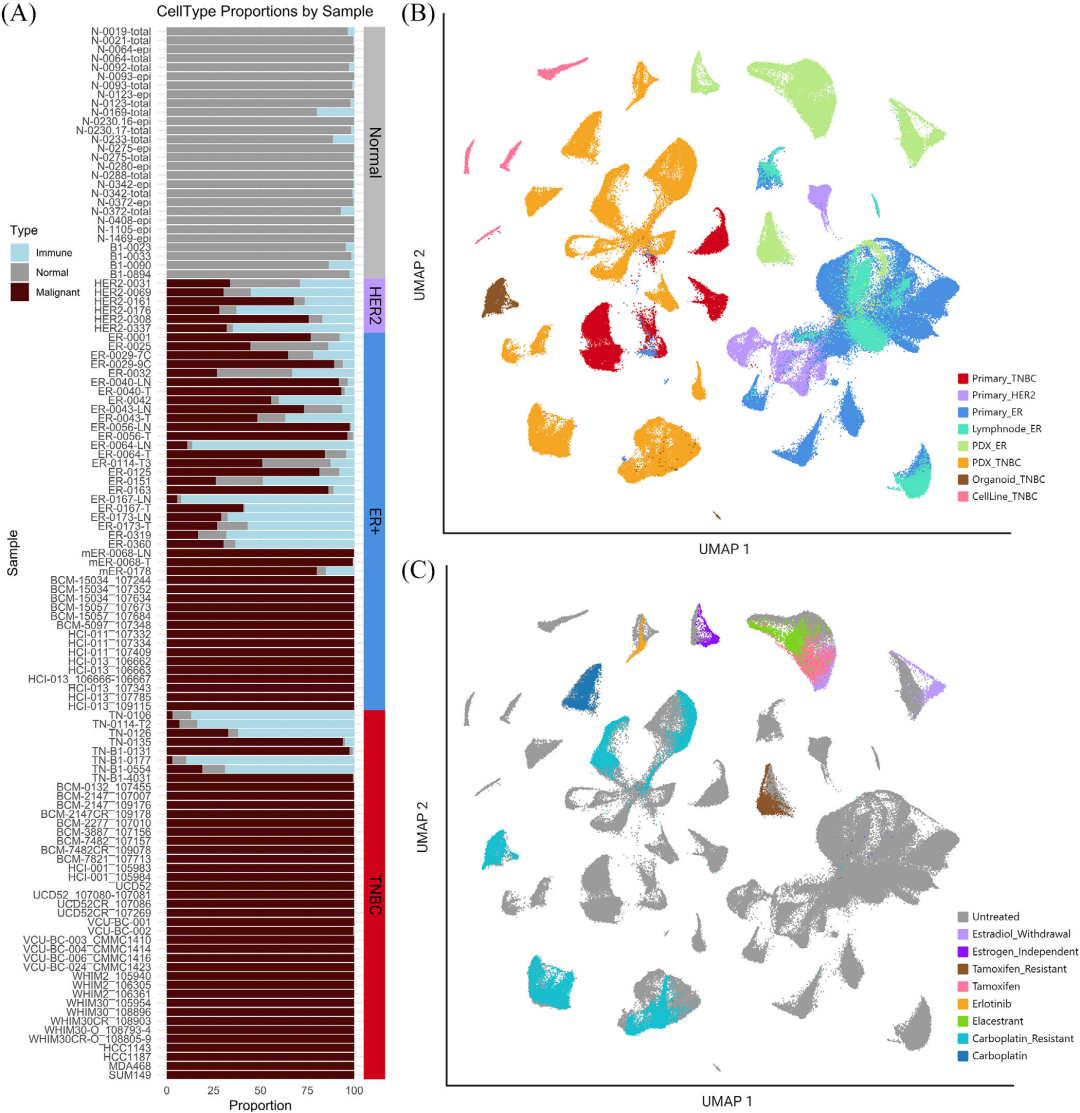
Cancer‐only clustering following removal of immune and normal cell clusters. (A) Per cent bar graph showing the proportions of immune (blue), normal (grey), and malignant (garnet) cell types in each sample. Clinical type is annotated by the bar along the right of the graph. UMAP visualisations of cancer‐only cell dataset coloured by (B) tissue of origin and clinical subtype and (C) treatment or condition.

In TNBC samples we observed a trend towards higher percentages of immune cell infiltrates in primary tumours when compared to ER+ and HER2+ primary samples (Figure 4A). This is not surprising as TNBCs have been previously annotated to contain greater amounts of tumour infiltrating lymphocytes and immunotherapies have shown greatest efficacy in this subtype, while ER+ tumours are generally considered immunologically ‘cold’.^32^, ^33^, ^34^ Correspondingly, upon normal cell removal some TNBC samples (most notably TN‐106 and TN‐0114‐T2, with 29 and 106 cells respectively) had relatively few tumour cells remaining.

To further assess malignant cell types, the resulting cells following immune and normal exclusion were merged into a combined dataset of 260 500 cancer cells, representing clinically typed ER+, HER2+, and TNBC. The resulting dataset of malignant‐only cells underwent normalisation, scaling, dimensional reduction and was visualised by UMAP projections annotated by clinical type and model (Figure 4B). Again, TNBC and ER+/HER2+ samples clustered distinctly away from one another, primarily on the left and right sides of the UMAP respectively, except for ER‐0319 which clustered with TNBC clinically typed samples (Figure 4B). The majority of primary HER2+ samples were again seen to cluster with a subset of ER+ primary samples. Of note, TNBC PDXOs clustered distinctly away from their founder PDX counterparts, suggesting distinct transcriptional features of this model system, deserving of further analysis.

### 2.4 Treated PDX samples exhibit molecular profiles similar to their untreated pair

We further sought to stratify our data based on applied treatment. Several PDX samples within this dataset have applied treatment and untreated isogenic paired samples. Of note, all 69 primary patient samples have been characterised as treatment naïve.^26^ Treated PDX samples exhibited overall transcriptional profiles that closely resembled their untreated paired samples, as indicated through their close proximity to their untreated isogenic pairs in the UMAP projection (Figure 4C). The main exception to this was noted to be ER+ PDX pairs under estradiol withdrawal conditions, an observation worthy of further study. The proximity of isogenic pairs in most contexts suggests that PDX models remain more transcriptionally like their matched untreated isogenic counterparts than to other treatment samples. Prior studies saw similar conservation of essential molecular features in patient tumour cells following treatment with therapeutic agent.^8^, ^9^ Within PDX models, this trend held true even in the case of developed resistance to carboplatin due to long term exposure to the drug over serial passages, where we note resistance status did not significantly shift clustering of cells.

This finding underscores the robustness and reliability of PDX models in retaining essential molecular features even after exposure to therapeutic interventions. Furthermore, this recapitulates trends in expression previously seen in patient tumour cells. This observation holds significant implications for translational research and preclinical studies, as it suggests that the robust molecular characteristics of tumour cells may remain largely unchanged even in the presence of therapeutic interventions. The congruence in molecular profiles between treated and untreated PDX samples highlights the need for differentially expressed gene analysis in evaluating treatment responses and deciphering the molecular intricacies associated with mechanism of resistance.

### 2.5 Transcriptional changes underlying resistance to platinum‐based chemotherapeutics in TNBC

**FIGURE 5:**
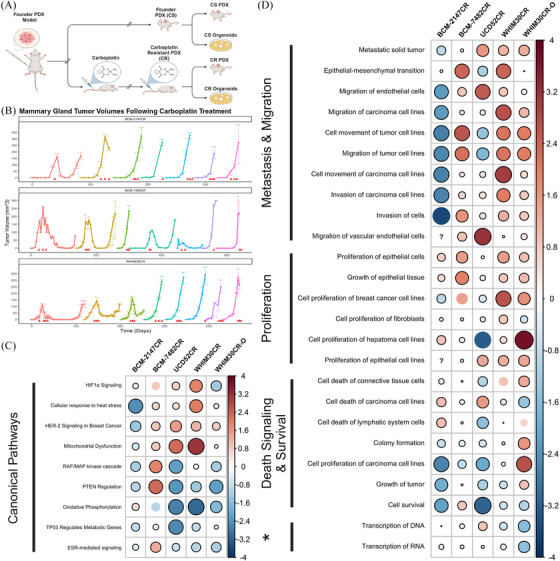
Transcriptional changes underlying resistance to platinum‐based chemotherapeutics in TNBC models. (A) Workflow schematic demonstrating development of carboplatin resistant (CR) and carboplatin sensitive (CS) pairs. (B) Tumour volume graphs for 3 PDX models contained within (BCM‐2147, BCM‐7482, WHIM30), starting with cells from founding PDX and monitored over serial passage with applied carboplatin treatments. Red arrows indicate the administration of carboplatin via intraperitoneal injection at dosage 40 mg/kg. Each serial passage of cells into new mice is represented by a new segment and colour on the larger parent graph. Of note: final segment of BCM‐7482CR graph represents data from the same cohort as sample BCM‐7482CR_109078. (C, D) Canonical pathways and disease/function annotations differentially regulated in CR models as observed through IPA analysis. The size and colour of the circle represent the z‐score associated with the pathway in that model comparing CS and CR pairs, positive values (blue) indicate activation in CR models, negative values (red) indicate inactivation or downregulation. Statistically significant associations (*p*  <  .05) are shown by a black border surrounding the circle. ‘?’ indicates insufficient data for *z*‐score calculation. Generated using the corrplot() function from the corrplot package. * = ‘Alterations in Transcriptional Programming’.

A major strength of this dataset lies in its incorporation of isogenic models of resistance. To understand how treatment effects gene expression at a single‐cell resolution, we generated resistance models by subjecting a subset of the parental PDX model to successive tumour passages coupled with the administration of carboplatin (40 mg/kg), a platinum‐based DNA intercalating chemotherapeutic (Figure 5A).^35^ Several models were inferred to be carboplatin resistant (CR) when no discernible reduction in tumour volume was observed following treatment compared to time‐matched carboplatin sensitive (CS) founder models (Figure 5B).

To identify transcriptional changes that occur with developed resistance, we performed differentially expressed gene (DEG) and pathway analysis on each set of paired CR and CS models. Pathway analysis involved scrutinising the significantly enriched pathways for potential overlap between model systems (Figure 5C and D). This approach allowed us to unravel the intricate molecular mechanisms underpinning resistance across diverse PDX/PDXO model pairs, providing valuable insights into the common pathways associated with chemotherapeutic resistance. Pathways involved in activation of HIFα, HER‐2 signalling, cellular response to heat stress, and mitochondrial dysfunction were significantly activated in 4 of the 5 CR models when compared to CS (Figure 5C). Furthermore, a number of canonical pathways were found to be inactivated in CR models, such as PTEN signalling, oxidative phosphorylation, and ESR‐mediated signalling.

Among those pathways associated with disease and function, several similarly associated and overlapping pathways were observed between CR models (Figure 5D). Many signatures associated with metastasis and migration were seen to be elevated broadly across these models. Curiously, BCM‐2147 showed slight activation of epithelial‐mesenchymal cells, but generally inactivation of other signatures associated with metastasis and migration. These findings suggest that broadly, CR models may have higher metastatic phenotype, excepting BCM‐2147. Cellular proliferation related signatures were also seen to be activated within many CR models. This aligns our observation that CR models tended to reach maximum tumour burden in fewer days than their CS pair. Interestingly, we concurrently saw activation of death signalling and inactivation of signatures of a small subset of proliferation and colony formation signatures across CR models. This nuanced observation may point to increased rates of cell turnover in CR models. Notably, we have seen higher instances of necrosis at the centre of some CR PDX models comparative to their CS pair. These findings taken together may suggest that larger CR tumours may experience rapid proliferation on the tumours outer expanding surface while more internal cells lack adequate nutrients resulting in increased death signalling. Additionally, we see activation of pathways linked to alterations in transcriptional programming such as those related to transcription of DNA/RNA, unsurprising given the mechanism of action associated with the drug carboplatin.

### 2.6 Distinct gene expression alterations underlay PDXO culture

**FIGURE 6:**
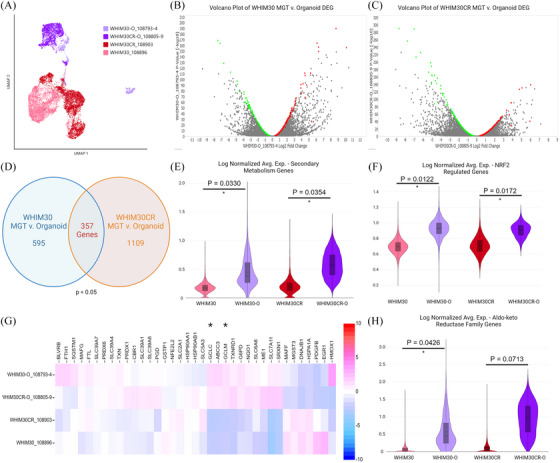
Differential gene expression analysis of time matched PDX MGT and PDXO in WHIM30 and WHIM30CR. (A) UMAP of reclustering of PDX and PDXO models included in analyses. Volcano plots of differentially regulated genes in (B) WHIM30 PDXO compared to PDX MGT and (C) WHIM30CR PDXO compared to PDX MGT. Genes selected for analysis highlighted in green (downregulated) and red (upregulated), genes were excluded with *p*‐values > .05 or low average read counts (defined by an average occurrence less than 1 count per cell across the dataset). (D) Venn diagram of overlapping gene count between model sets. Violin plots of log normalised average expression for genes within signatures for (E) secondary metabolism genes, (F) genes regulated by NRF2, and (H) aldo‐keto reductase family genes. *p* Values from unpaired *t*‐test. (G) Heatmap visualisation of NRF2 pathway genes as log2 fold change between samples. Glutamate‐cysteine ligase modifier (GCLM) and catalytic subunits (GCLC) annotated with *.

As previously noted, this dataset includes time matched, batch sequenced, PDX and PDXO samples from the founder lines WHIM30 and WHIM30CR (Figure 6A). Remarkably, PDXO and PDX samples from the CS and CR lines do not cluster by resistance status, but rather by model system. WHIM30 and its isogenic pair WHIM30CR fall within the same cluster by UMAP, having globally similar transcriptional profiles, however, PDXO pairs did not cluster with their originating PDX models, despite being seeded from the same time‐matched cells (Figure 6A). This finding is contrary to previous reported comparisons of PDX and PDXOs bulk tissue RNA sequencing.^36^ DEG analysis uncovered 595 significant (*p* < .05) genes between PDX/PDXO samples in WHIM30 founder line, and 1109 significant genes differentially expressed between PDX/PDXO model types in WHIM30CR following Benjamini‐Hochberg multiple testing correction (Figure 6B and C). Of these, 357 genes were seen to be differentially expressed in both sets in the same direction (Figure 6D). These 357 genes were then evaluated via gene set enrichment analysis to determine pathways of interest. Secondary metabolism genes were found to be significantly differentially regulated between PDX/PDXO sets with generally higher expression seen in PDXO samples, suggesting alterations in cellular metabolism profiles (Figure 6E). Additionally, there was found to be significant upregulation of genes associated with the NRF2 pathway in PDXO samples. Namely genes both involved in this pathway and regulated by the NRF2 transcription factor were seen to be upregulated in organoid models (Figure 6F and G). Interestingly, among transcripts most upregulated in PDXOs were glutamine‐cystine ligase catalytic and regulatory subunits GCLC, GCLM (Figure 6G). Several aldo‐keto reductase family genes were likewise seen among those most upregulated in PDXO models (Figure 6H). These findings taken together suggest that PDXOs have distinct metabolic profiles when compared to their PDX counterparts, suggesting possible non‐canonical glutamate‐cysteine ligase activity as was previously seen in cancer cell lines.^37^ These changes in metabolic function underlying organoid culturing, have serious implications for drug response and in vitro testing.

### 2.7 PAM50 pseudo‐bulk comparison with clinically typed sample annotations

**FIGURE 7:**
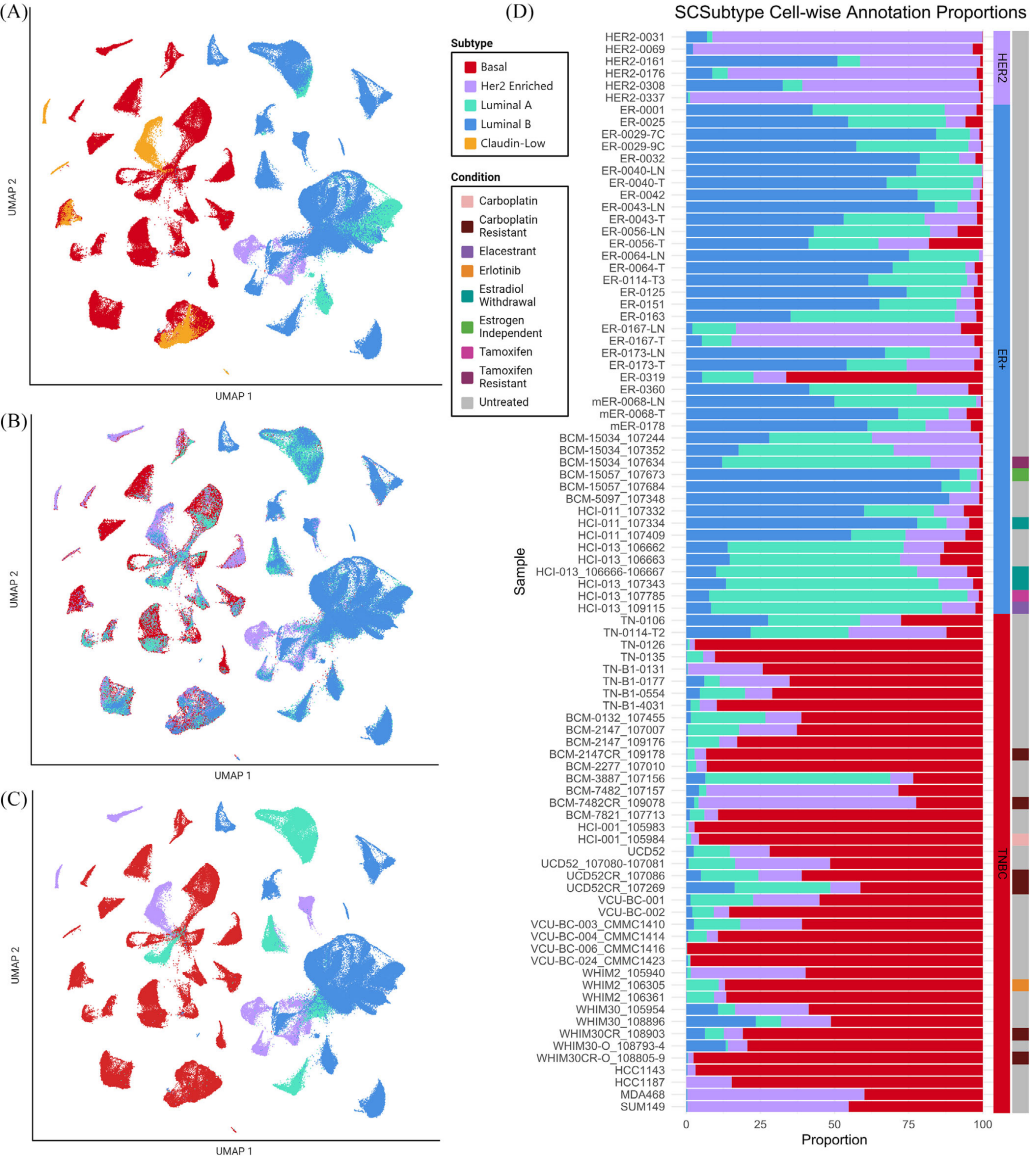
SCSubtype single‐cell typing methodology on mixed cancer‐only set. (A) UMAP visualisations of cancer‐only cell dataset coloured by sample‐wise pseudo‐bulk PAM50 and claudin‐low centroid predictors. (B) UMAP visualisation of SCSubtype cell‐wise annotations. (C) UMAP visualisation of SCSubtype sample‐wise annotations, as denoted by majority call. (D) Bar graph showing proportion of cells annotated as each of the 4 molecular subtypes classified by SCSubtype, ordered by clinical subtype and model type. Top to bottom: Her2‐enriched primary, ER+ primary, ER+ PDX, TNBC primary, TNBC PDX/PDXO, TNBC cell line. Conditions displayed for each sample in the right‐most bar.

To further interrogate the molecular portraits of samples within our dataset, we created pseudo‐bulk RNA profiles for each sample in order to apply the PAM50,^8^, ^9^, ^10^, ^11^, ^38^ and claudin‐low centroid predictors (Figure 7A).^39^ Intriguingly, some PAM50 classifications did not align with expected calls based on clinical subtyping, such as in the case of ER‐0319, clinically typed as PR+/ER^low^ via IHC but annotated as ‘basal‐like’ by the PAM50 predictor, a molecular subtype most closely associated with TNBCs.^8^ While initially surprising, recent literature notes molecular similarities between TNBCs and ER–/PR+ breast cancers.^40^ Additionally, it has been shown that a subset of ER^low^ cancers (1–9% positivity) cluster primarily with basal‐like samples in gene expression profiling.^41^, ^42^, ^43^ Further incongruencies with PAM50/claudin‐low and clinical‐type included TN‐0106 and TN‐0114‐T2 clinically classified as TNBC were unexpectedly labelled as ‘luminal‐A’ and ‘luminal‐B’ respectively, by PAM50 predictor. This difference may be attributed, to the 15–20% of TNBCs which are molecularly classified as luminal androgen receptor (LAR) positive,^15^, ^44^, ^45^ or to the low percentages of remaining malignant cells post immune/normal cell removal within these samples, underscoring the importance of considering multiple factors in validating subtype classification. HER2+ clinically typed primary samples were typed by PAM50 centroid predictor as similarly HER2‐enriched (HER2E), excepting HER2‐0161 and HER2‐0308 samples which were typed ‘luminal B’. This is not surprising due to the close clustering of these samples with other luminal‐like or clinically ER+ samples. Interestingly, several samples were annotated as claudin‐low by this pseudo‐bulk methodology, a molecular subtype not often differentiated in the clinical setting. Claudin‐low prediction was applied independent of PAM50 centroid predictor, and all claudin‐low tumours in this sample set were initially typed as basal‐like; this is not surprising given the majority of claudin‐low samples are ER–/PR–/HER2– and the clinical subtype TNBC is thought to be primarily composed of basal‐like and claudin‐low subtypes.^46^, ^47^

Among those typed claudin‐low were PDXs (BCM‐7482_107157, BCM‐7482CR_109078, WHIM30CR_108903), PDXO (WHIM30‐O_108793‐4), and cell lines (HCC1143, SUM149). The inclusion of PDX sample WHIM30CR_108903 and PDXO sample WHIM30‐O_108793‐4, as claudin‐low here were surprising as the founder PDX WHIM30 (represented here by WHIM30_105954 & WHIM30_108896) and corresponding resistant PDXO (WHIM30CR‐O_108805‐9), were categorised as basal‐like. Previous studies have noted that claudin‐low characteristics can increase in samples post treatment with neoadjuvant chemotherapies^47^ (such as with WHIM30CR_108903); however, admittedly by this mechanism alone, it is peculiar why WHIM30‐O_108793‐4 was typed claudin‐low and not its carboplatin resistant counterpart (WHIM30CR‐O_108805‐9). HCC1143 and SUM149 cell lines were previously characterised as containing populations of claudin‐low cells,^48^ so it is unsurprising that they would be among the few samples typed as claudin‐low within this dataset. Likewise, BCM‐7482 founder and resistant PDX models were both typed to be claudin‐low by this predictor, which aligns with prior characterisations of this model within our lab.

### 2.8 Intrinsic subtyping analysis of scRNA‐seq in human breast cancer cells further defines molecular heterogeneity between model systems

In order to evaluate the molecular heterogeneity present within the malignant cell subset, we performed intrinsic molecular subtyping (basal, luminal A, luminal B, and HER2‐enriched) utilising SCSubtype methodologies.^10^, ^49^ The results from SCSubtype analysis highlight the inherent heterogeneity within breast cancer samples. The cell‐wise annotations provide a comprehensive snapshot of the diversity in molecular subtypes, shedding light on the intricate interplay of genetic signatures within the cancer‐only dataset. Cell‐wise subtype annotations allowed us to see variation heterogeneity within a sample (Figure 7B), while sample‐wise subtype calls based on majority cell calls within a sample allowed us to address concordance with PAM50 subtyping (Figure 7C).

Of note, we saw ∼70% concordance with calls from PAM50 pseudo‐bulk analysis, which outperformed when compared to the 66% concordance seen with pseudo‐bulk in the original testing‐set.^10^ However, in ER+ cohorts we noted more incongruencies with respect to Luminal A versus Luminal B calls between PAM50 predictor and SCSubtype majority call, noting ∼61% concordance with PAM50 pseudo‐bulk analysis in ER+ clinically typed samples. However, SCSubtype analysis consistently classified ER‐0319 as ‘basal‐like’ via the majority call, echoing findings from prior PAM50 predictors. This unexpected alignment with the basal subtype suggests that, despite the initial IHC characterisation, ER‐0319 exhibited molecular features more closely resembling basal‐like samples. TNBC models BCM‐3887 and BCM‐7482 which by majority call were typed Luminal A and HER‐2 enriched respectively, however classified by PAM50 and claudin‐low centroid predictors, were typed basal‐like and claudin‐low respectively. Interestingly, claudin‐low subtype is not included in SCSubtype methodologies; however, both the CS and CR pair in BCM‐7482 PDX and SUM149 cell line were typed HER2E by majority SCSubtype cell‐wise call and claudin‐low within the prior pseudobulk analysis. This alignment of three claudin‐low samples with HER2E subtype, suggests there may be overlap between claudin‐low subtype and cell‐wise calls for HER2E subtype under SCSubtype methodologies. It was noted that all TNBC models, which were not typed basal‐like by majority call following SCSubtype analysis, contained a subset of basal‐cells which constituted the second most abundant cell type in these samples.

Although the overall gene expression profiles of PDX models did not exhibit global shifts with treatment, except in estradiol withdrawal (EWD) conditions, intriguing observations emerged regarding the influence of certain treatments on the proportions of single‐cell subtypes in both PDX and PDXO models (Figure 7D). A notable trend was observed in the proportion shifts towards more basal cells in time matched WHIM30, WHIM30‐O (PDXO), BCM‐2147, and UCD52 CR models compared to their isogenic CS counterparts. This nuanced effect can likely be attributed to the fact that subtype signatures primarily focus on the expression of cancer‐related pathways, which could be markedly influenced by targeted therapeutic agents. Conversely, a contrasting trend was noted when comparing CR and CS pairs in BCM‐7482, which by majority call was typed HER2E. Of note, an expansion of the HER2E population of cells is seen in this CR sample when compared to the previously sequenced CS pair. Thus, in each CR/CS pair we see expansion of the majority cell type within each CR model comparatively. This state‐specific observation indicates predictive utility of these shifts in subtype calls for anticipating drug response.

### 2.9 Profiling of ER+ malignant cells reveal insights into estrogen‐dependent responses

**FIGURE 8:**
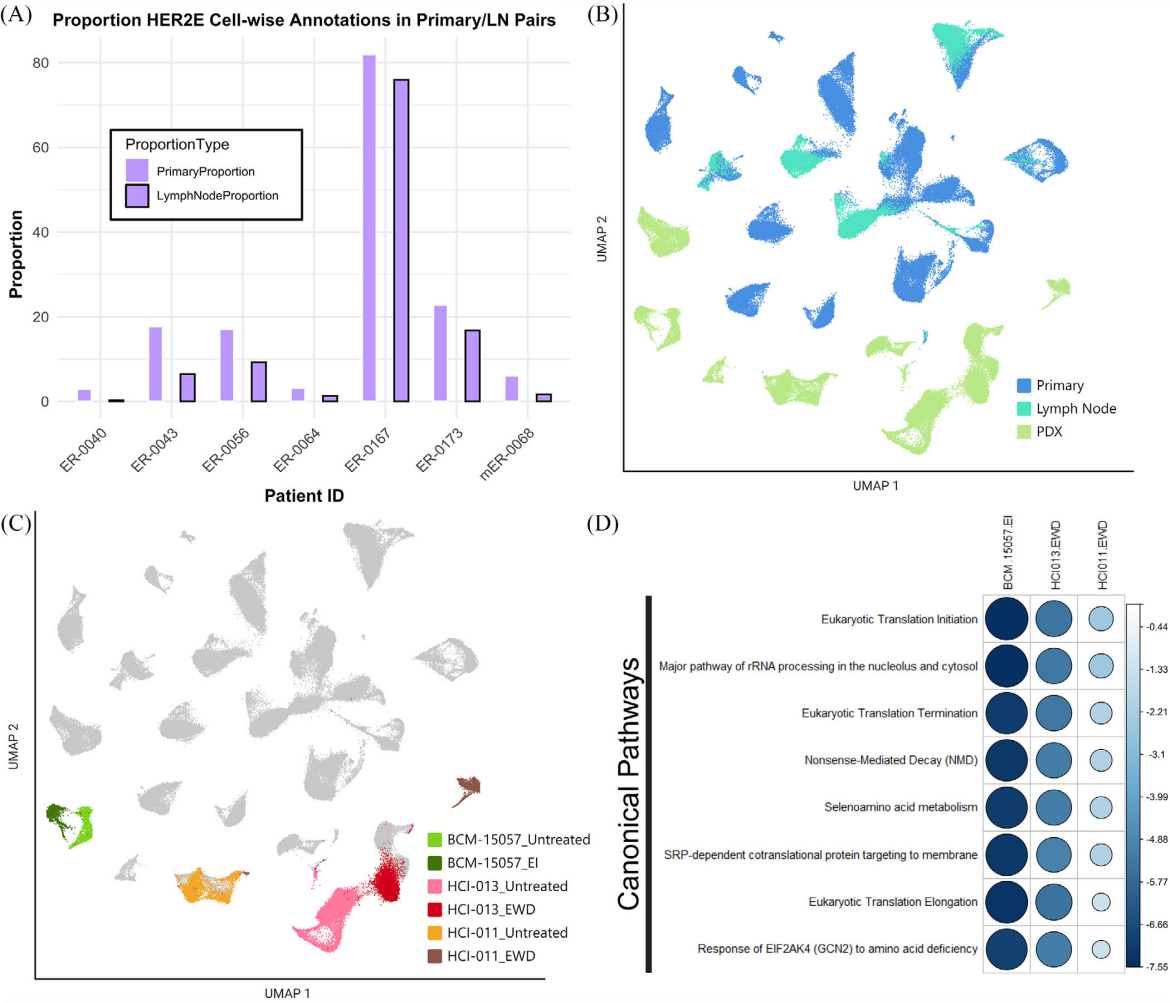
Transcriptional profiles of ER+ malignant cells reveal model differences. (A) Proportion of HER2‐enriched cell‐wise calls via SCSubtype in matched patient primary and lymph node metastasis. (B) UMAP of ER+‐only subset coloured by model/tissue type. (C) UMAP of ER+‐only subset, coloured by PDX and treated condition. (D) Canonical pathways differentially regulated in estradiol withdrawal (EWD) and estrogen independent (EI) conditions as compared to E2‐treated (untreated) or wild‐type models. The size and colour of the circle represent the *z*‐score associated with the pathway in that model compared with the untreated pair, positive values (blue) indicate activation in EWD models, negative values (red) indicate inactivation or downregulation. Statistically significant associations (*p*  <  .05) are shown by a black border surrounding the circle (all). Generated using the corrplot() function from the corrplot package.

When evaluating subtyped proportions between sample types, we noted a trend in HER‐2 enriched aligning cell proportions between matched primary and lymph node samples (*p*.adj = .0625). Lymph node samples show a trend towards decreased proportions of cells aligning with this subtype when compared with matched primary tissues (Figure 8A). No trends were noted for other subtypes between lymph node metastasis and primary tissues.

This finding prompted further examination of the ER+ clinically typed samples within the dataset. To facilitate this analysis, UMAP visualisations and clustering were generated for malignant cells in the clinically ER+ subset of samples (Figures 1A and 8B). We observed that individual patient samples formed more distinct clusters than seen in the mixed subtype set, with matched lymph node samples clustering alongside the primary sample from that patient (Figure 8B). Interestingly, PDX samples were observed to cluster near the bottom of the UMAP visualisation, not interspersed with the direct from patient samples, suggesting differences in the overall transcriptional landscape between these sample types.

To further assess samples in this cohort, clustering of cells from isogenic PDX pairs was visualised, those either given subcutaneous β‐estradiol pellets (E2‐treated, denoted in Figure 8C as ‘Untreated’), and those which underwent EWD after tumours developed, including BCM‐15057_EI which had developed an estrogen independent (EI) phenotype under EWD conditions (Figure 8C).^50^ Similarly, to what was observed in the mixed sample set, ER+ samples which had been grown under EWD conditions exhibited significant shifts in transcriptional profiles when compared to their E2‐treated pair, likely due to the role of estrogen and the ER in transcription.^51^, ^52^, ^53^, ^54^, ^55^, ^56^ Markedly, EWD conditions caused a considerable shift the HCI‐011 cells relative to the E2‐treated population. Conversely, shifts between BCM‐15057 EI and E2‐treated as well as HCI‐013 EWD and E2‐treated populations displayed less dramatic shifts in cell clustering when comparing EWD and E2 treatment conditions, demonstrating more relative stability across these samples. This may be due to mutational ESR1 within these samples as HCI‐013 has been previously annotated to contain an ESR1 Y537S mutation (1610A > C), and similarly BCM‐15057 EI has an acquired Y537S mutation, which leads to constitutive activation of ER.^50^, ^57^ Our findings of less dramatic shifts between these pairs are consistent with previous reports that Y537S mutation specifically results in similar principal components when stimulated with E2 or under hormone deprivation conditions.^58^

To further interrogate transcriptional changes underlying EWD conditions, we performed DEG and pathway analysis in EWD/E2‐treated pairs. Although two of the three estrogen withdrawn models harbour Y537S mutations to ESR1, all three pairs show decreased translation‐related transcripts under EWD conditions (Figure 8D). The differentially expressed pathway trends observed in this mixed set of mutant and normal ER, between EWD and E2 conditions, may be uniquely regulated by liganded receptor and not stimulated by constitutively active mutant ER.

### 2.10 Multisubtype analysis of TNBC models reveal subtype strengths

**FIGURE 9:**
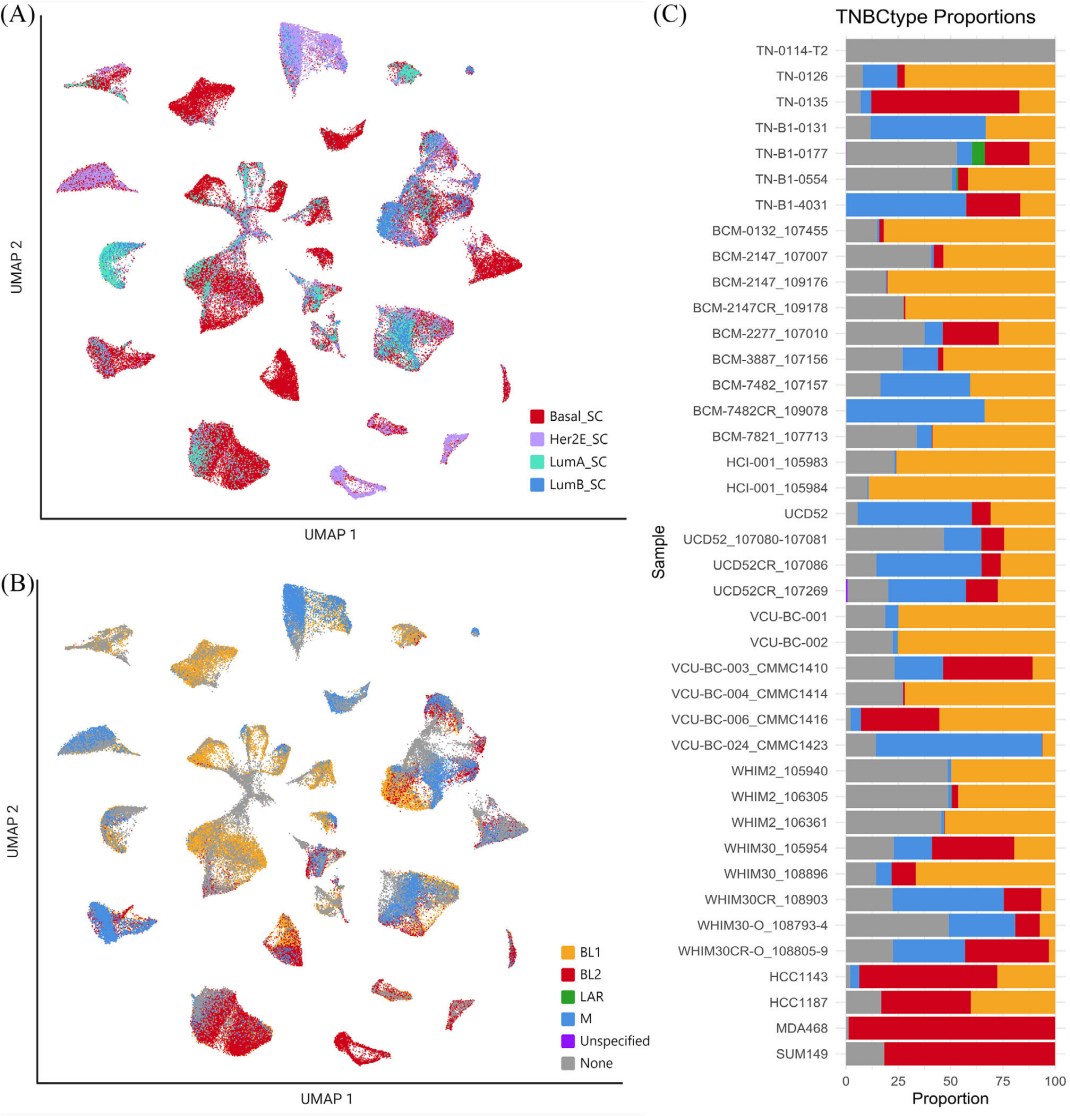
Subtyping comparison in TNBC cancer cell subset. (A) UMAP visualisation of SCSubtype cell‐wise annotations projected onto TNBC‐only subset. (B) UMAP visualisation of TNBCtype cell‐wise annotations. (C) Bar graph showing proportion of cells annotated one of the 4 molecular subtypes classified by TNBCtype, ordered by clinical subtype and model type. Top to bottom: TNBC primary, TNBC PDX/PDXO, TNBC cell line. Of note, ‘Unspecified’ denotes cells, which were not positively correlated with one of the 4 subtypes, and ‘None’ represents those cells which were untyped due to missingness in gene expression.

In addition to the four molecularly intrinsic subtypes of breast cancer, previous studies have defined four molecular subtypes within TNBCs. To validate and compare these subtype calls within our clinically typed TNBC samples, we generated a data subset of clinically typed TNBC samples (Figure 1B). We then visualised the heterogeneity within these new clusters as previously annotated by SCSubtype proportion (Figure 9A). Individual cells were then subtyped by centroid correlation to one of the 4 TNBC subtypes, including Basal‐like 1 (BL1), Basal‐like 2 (BL2), Luminal androgen receptor (LAR), and Mesenchymal (M) subtypes (Figure 9B).^15^, ^16^ These subtyping annotations allowed us to look further at the heterogeneity within TNBC samples.

Some cells were excluded from subtyping due to insufficient transcriptional profiles. Two samples, TN‐0106 and TN‐0114‐T2, were excluded due to their limited cell numbers after immune and normal cell filtering. TNBCtype cell‐wise calls did not exhibit significant stratification based on any SCSubtype classifications. We noted that many cells from our cell line samples were primarily correlated with BL2 subtype (with two models composed of mixed BL1 an BL2), while PDX and primary cells were primarily correlated with BL1 and M subtypes. Notably, few overall cells were found to be associated with the LAR subtype, which likely reflects the rare nature of this subtype overall in TNBC (Figure 9C).^59^ As seen with SCSubtype calls, cell‐wise annotations of TNBCtype are seen to shift in proportion with applied treatment, with an expansion of M aligning cells seen in some CR models (BCM‐7482, WHIM30, and time‐matched UCD52), however it was unclear if this is representative of the natural variation within our sample set. This analysis, however, was limited due to the inability to subtype a proportion of cells within samples.

### 2.11 Therapeutic drug efficacy stratification based on cell‐wise subtyping analyses in PDX models

To assess the utility of subtyping methods as predictive tools for therapeutic response, we integrated drug screening data for 555 anti‐cancer compounds (NCI NExT Oncology Interrogation Tools Library)^60^ targeting cancer‐relevant pathways across 18 TNBC and 5 ER+ clinically typed PDX models with projected proportions of cells categorised by SCSubtype and TNBCtype subtyping methodologies. This analysis utilised these subtyping methodologies at the single‐cell level to estimate the proportion of subtype‐aligning cells within each PDX model. Drug screens were performed on single‐cell suspensions from PDX mammary gland tumour digestions. Cell viability was measured following 3‐day treatment with therapeutic agents as previously described.^50^, ^61^, ^62^ These projections were subsequently applied in order to model responses to *ex vivo* drug screening, investigating whether these subtypes serve as meaningful indicators for the response to specific classes of compounds.

### 2.12 SCSubtype proportions predict drug response in mixed set of ER+ and TNBC PDX models

**FIGURE 10:**
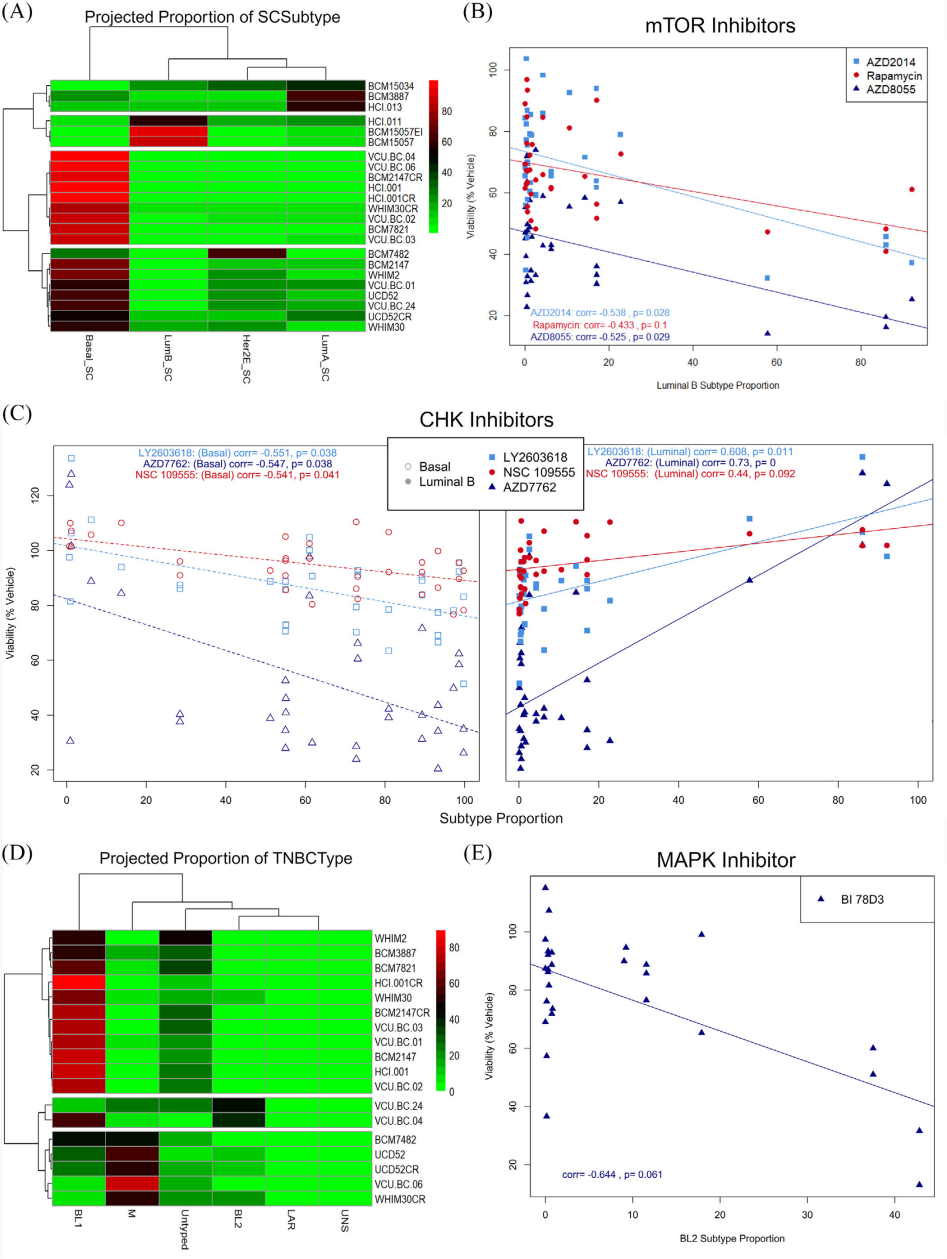
Integrative analysis of cell‐wise subtyping with high throughput drug screening data. (A) Projected proportions of SCSubtype cell‐wise call onto relevant PDX models. (B) Scatter plot of cell viability following treatment with 3 mTOR inhibitors as a per cent of vehicle treated cells given varied luminal B subtype proportions. (C) Scatter plots of cell viability following treatment with 3 CHK inhibitors as a per cent of vehicle treated cells given varied basal (left) or luminal B (right) subtype proportions. (Correlation values and adjusted *p*‐values given for each drug). (D) Projected proportions of TNBCtype cell‐wise call onto relevant PDX models. (E) Scatter plot of cell viability following treatment with MAPK inhibitor (BI 78D3) as a per cent of vehicle treated cells given BL2 subtype proportions.

First, we examined the distribution patterns of SCSubtype annotations within each PDX by projecting the proportions of subtype calls onto relevant PDX models (Figure 10A). For this analysis we utilised both ER+ and TNBC PDX models for which we had SCSubtype information available. Where multiple untreated samples were present, the averages of the proportions were taken for projection onto the necessary PDX models. Through hierarchical clustering, we observed four main groups based on these projected proportions; those clusters could be best defined by their luminal and basal proportions (Luminal A high‐intermediate, Luminal B high, Basal high, and Basal intermediate). A significant constraint identified in this dataset was the low proportion of HER2E typed cells in the PDX samples.

Following subtype proportion projection, correlation analysis was performed between the projected proportions of each subtype and the viability of cells following treatment with anti‐cancer compounds given at 1 μM concentration. In this context, negative association with a subtype denotes increased responsiveness to the drug given more abundance of the cell subtype; while positive associates denote decreased responsiveness of the subtype following drug treatment. This analysis returned several suggestively (*p*.adj < .1) and significantly (*p*.adj. < .05) associated therapeutic agents for Basal and Luminal B subtypes, and one association with Luminal A subtype (Table S2). HPI‐1, a drug targeting hedgehog signalling, was negatively correlated with abundance of Luminal A cells, suggesting lower viability of cells following treatment in samples with more Luminal A cells. Hedgehog pathway is known to be involved in breast cancers and previously annotated to be more associated with luminal types.^63^

Interestingly, we observed significant negative correlation with Luminal B subtype and decreased viability following treatment with several mTOR inhibitors (AZD2014, AZD8055, and Rapamycin) in samples which were more abundant for this subtype (Figure 10B). This was surprising as this pathway has been previously characterised as more upregulated in a subset of basal models.^64^, ^65^ This finding may represent a therapeutic vulnerability in luminal malignancies to inhibition of this pathway.

Of note 14 drugs showed significant association with both Luminal B and Basal subtypes in opposite directions, demonstrating the utility of SCSubtype methodologies when used to stratify samples in a mixed set into intrinsic subtypes which are informative for therapeutic response. When splitting this analysis into two sets based on ER+ status, we saw significantly fewer overall associations, indicating that the predictive ability of this subtyping methodology is greatly enhanced by a mixed set of samples. In the case of drugs which were associated with both Basal and Luminal B subtypes, all were positively associated with Luminal and negatively associated with the Basal subtype, suggesting that basal‐like cells are more sensitive to these drugs. Among those in this category were several CHK inhibitors (AZD7762, LY2603618 and NSC 109555) and two aurora kinase inhibitors (AT9283 and ZM 447439), both pathways currently under investigation in TNBCs due to their activation in these models (Figure 10C).^66^, ^67^, ^68^, ^69^, ^70^, ^71^ Interestingly, two Bcl‐2 inhibitors [Obatoclax Mesylate (GX15‐070) and ABT737] were seen to be negatively associated with Luminal B subtype, while one (Bax channel blocker) was conversely seen to be negatively associated with Basal and positively associated with Luminal B subtypes. The contraindication of drugs targeting this pathway are curious in these models. Bcl‐2 was previously shown to be overexpressed in ∼85% of ER+ breast malignancies, and Bcl‐2 positivity has been associated with poor clinical outcome in TNBC models, suggesting possible therapeutic vulnerabilities within both luminal and basal subtyped tumours.^72^, ^73^ Indeed, these findings may suggest that certain Bcl‐2 inhibitors may have subtype specific activity, while both subtypes may have vulnerability to this pathway.

### 2.13 TNBCtype cell‐wise annotations demonstrate predictive response value in TNBC subset

When examining the proportion patterns of TNBCtype cell‐wise subtype calls within our TNBC PDX models, a notable prevalence of Basal‐like 1 (BL1) cells was noted in the dataset. Hierarchical clustering of our 18 included TNBC PDX models based on their projected TNBCtype proportions revealed four main clusters primarily driven by their proportions of BL1, BL2, and M subtypes (BL1 high‐intermediate, BL2 intermediate, M high‐intermediate) (Figure 10D).

Subsequently, we explored correlations between proportion of TNBCtype subtype and drug responses at 1 μM concentrations. Notably, we observed significant correlations between several agents and the projected proportion for several subtypes (Table S3). BL1 subtype was positively correlated with KD 5170, an agent targeting histone deacetylases class I and II. Indeed, some HDAC inhibitors have shown promise in treatment of TNBC.^74^, ^75^ BL2 subtype was seen to be negatively correlated with two drugs targeting RAF kinase and MAPK, both previously identified as targetable in basal malignancies (Figure 10E).^76^, ^77^, ^78^ Furthermore, BL2 subtype was seen to be positively correlated with one drug targeting JAK pathway, suggesting this subtype of TNBC may be less responsive to treatment with JAK pathway inhibitors than other subtypes. M proportions were positively correlation with a MEK inhibitor (PD0325901). Indeed prior studies found that the M subtype could be effectively treated by targeting MEK/MAPK pathway.^79^, ^80^

Of particular interest, the subtype seen to be predictive of response to the most therapeutic agents was the LAR grouping. This subtype had relatively few cells overall. In fact, only 3 PDX models had any LAR typed cells and among them, all had projected proportions of less than .2%. Among the many associations noted to be significantly correlated with proportion of this subtype, drugs targeting mTOR, EGFR, and PI3K were all seen to be negatively correlated. This finding is not surprising, as majority of LAR tumours have activating PIK3CA and AKT mutations and have been shown to be sensitive to these agents.^45^, ^81^, ^82^, ^83^ Interestingly, many of the drugs correlated with LAR subtype were previously seen to be similarly associated with Luminal B subtype in our SCSubtype analysis, an unsurprising finding given the similarities of these two subtypes.

## 3 DISCUSSION

Our study provides a high‐resolution transcriptional atlas of human breast cancer cells, integrating a large‐scale comprehensive dataset comprising various sample types, treatments, and isogenic pairs of drug‐resistant/sensitive models. Many similar datasets focus on primary tumours and immune interactions, not allowing for comparison between model systems.^10^, ^84^ These important works, while providing crucial insights into the immune microenvironment of breast cancer, does not extend to metastatic or model systems like PDXs or organoids, which are crucial for studying therapeutic responses and drug resistance. Our findings demonstrate that PDX models more accurately reflect patient tumours' heterogeneity and cell cycle dynamics than cell lines, as previously noted in other works.^85^, ^86^ However, our work adds to this field by integrating PDX and PDXO models from isogenic lineages to analyse shifts in gene expression, challenging other works in which organoids were reported to faithfully recapitulate disease heterogeneity and phenotypes.^87^ Additionally, the distinct metabolic transcript profiles observed in PDXOs compared to PDX tumours highlight the limitations of relying solely on organoids for metabolic studies. Our multimodel approach, therefore, provides a broader perspective on the data presented in these studies; future studies should expansively assess how cancer cellular physiology changes when cells are grown in matrices and chambers as organoids or when growing as metastases spreading in vital organs.

This integration enabled us to visualise malignant, normal, and immune clusters within our samples. Noting that TNBC samples demonstrated a trend in higher percentages of immune cell infiltrates in primary tumours compared to ER+ and HER2‐amplified primary samples, aligning with the prior observations of robust immune infiltrates in this subtype.^88^, ^89^, ^90^ The heightened immune cell infiltrates in TNBC primary tumours echo prior findings of an immunogenic microenvironment, likely contributing to the responsiveness observed in TNBCs to immune modulatory agents. The unexpected alignment of ER‐0319 with the basal subtype raises intriguing questions about the underlying molecular features and potential therapeutic vulnerabilities that may have been overlooked in traditional clinical characterisations, emphasising the importance of molecularly intrinsic subtyping methods like SCSubtype or PAM50 for a more comprehensive understanding of tumour characteristics. This example highlights the limitations of relying solely on clinical markers to subtype and treat breast cancer, demonstrating the need for advanced molecular profiling techniques to unveil the intricate heterogeneity within samples for refined treatment strategies. Dual subtyping may be particularly important for tumours which are ER+ or ER^low^ by clinical typing and molecularly basal‐like, as these malignancies have been characterised to respond very differently to therapeutic agents than other clinically ER+ tumours.^91^ Moving forward, future advancements in personalised medicine may benefit from integrating both clinical protein markers and molecular subtypes to optimise treatment selection.

Of note, UMAP visualisations and cluster analysis allowed for the examination of model heterogeneity, noting limited heterogeneity in cell lines compared to other model systems, suggesting these models less faithfully recapitulate the natural heterogeneity seen in human breast malignancies. We further stratified our data based on applied treatment, observing that treated PDX samples exhibited overall transcriptional profiles closely resembling their untreated paired samples, with notable exceptions observed in ER+ PDX pairs under estradiol withdrawal conditions. This finding underscores the robustness of PDX models in retaining essential molecular features after exposure to therapeutic interventions, aligning with trends seen in patient tumour cells and emphasising the importance of differentially expressed gene analysis for evaluating treatment responses and understanding mechanisms of resistance.

As previously noted, a major strength of this dataset lies in its incorporation of isogenic models of resistance, where the development of carboplatin resistance was observed in several TNBC PDX models. DEG and pathway analysis on paired CR and CS models, revealing common pathways associated with chemotherapeutic resistance across diverse PDX/PDXO model pairs. Activation of pathways linked to alterations in transcriptional programming, such as those related to DNA/RNA transcription, was notably observed, likely owing to the mechanism of action of carboplatin and other platinum‐based drugs.^35^, ^92^ In four out of five CR models, pathways related to HIFα activation, HER‐2 signalling, cellular response to heat stress, and mitochondrial dysfunction were significantly upregulated while several canonical pathways, including PTEN signalling, oxidative phosphorylation, and ESR‐mediated signalling, were found to be downregulated in CR models. Briefly, HIF‐1α and hypoxia pathways have been previously implicated as playing a role in drug resistance in TNBC models.^93^, ^94^ The implications of HER‐2 signalling in this context are deserving of further study, especially in BCM‐7482 where we note expansion of HER2E subtype in CR models compared to CS. Similarly, heat shock proteins have been studied as a significant element within the intricate and multifaceted reaction of cancer cells to platinum‐based chemotherapeutics but the implication of their expression is not well studied and may be context‐dependant.^95^ The upregulation of mitochondrial dysfunction pathway within these models is of particular interest for future study as mitochondrial dynamics in TNBC have been annotated to play important roles in metastasis and growth. Interestingly, PTEN has been implicated in altering repair processes during DNA damage response, so it is curious that CR models are associated with inactivation of this pathway.^96^, ^97^, ^98^ An intriguing observation was noted when taking these pathways together, mitochondrial metabolism plays key roles in cancer cell survival and proliferation, alongside this some solid tumours can thrive in less optimal oxygen available environments via mitochondrial dysregulation and hypoxia adaptive metabolic alterations which up‐regulate HIF‐1 factors.^99^, ^100^ We posit that under suboptimal oxygen conditions, the oxidative phosphorylation within these tumours may differ from normoxic conditions, leading to the observed downregulation of this pathway in CR models. Recognition of these metabolic alterations present possible vulnerabilities for future targeting of CR disease.

Several disease‐associated pathways were similarly activated among CR models, with heightened metastatic signatures observed broadly except in BCM‐2147, indicating a potential heightened metastatic phenotype associated with most CR models. Of note, BCM‐2147 appeared divergent in several major pathways, suggesting the mechanism of resistance in this model may be different. Additionally, concurrently increased cell necrosis and proliferation in many CR models may suggest rapid proliferation at the tumour periphery and nutrient deficiency internally, similar to what has been observed during tumour processing for some of these models. These findings underscore the complex interplay between tumour size, nutrient availability, and cell proliferation in carboplatin‐resistant models, suggesting potential avenues for targeting tumour microenvironments to enhance therapeutic efficacy and combat resistance mechanisms.

In addition to isogenic CR/CS pairs, we present here isogenic PDX and PDXO cultures. Our study uncovered distinct metabolic profiles in PDXOs compared to their PDX counterparts, with upregulation of aldo‐keto reductase family genes and genes related to NRF2 signalling. The metabolic changes underlying organoid culturing have significant implications for drug response and in vitro testing. These observations suggest a potential shift in cellular metabolism, possibly towards altered redox regulation. Furthermore, increased expression of GCLC and GCLM implicate possible alterations in glutamine‐cysteine ligase activity within PDXOs.^37^ As mentioned earlier, this indicates unique transcriptional characteristics within this model system, contradicting previous comparisons of bulk tissue RNA sequencing between PDX and PDXOs.^36^ However, a prior study showed that cell lines grown in vivo versus in vitro showed distinct metabolomic profile shifts similar to what our study notes in PDX versus PDXO cultures, suggesting that this may be a common divergence of in vitro conditions.^101^ These metabolic divergence may influence drug response mechanisms, introducing the notion that drug efficacy in PDXOs might be influenced not only by the tumour microenvironment but also by distinct metabolic adaptations. Considering the vital role metabolism plays in cancer cell survival, these findings prompt further exploration into the functional consequences of these metabolic shifts and their impact on the efficacy of therapeutic interventions. We speculate that deviations from the current standard for organoid culturing, may serve to improve the ability of this model to recapitulate in vivo conditions.

Subtyping methodologies, such as SCSubtype and TNBCtype, have contributed to our understanding of intrinsic heterogeneity within breast cancers, although their comparative strengths and applications across different model systems warrant further investigation. Building upon these advancements, subtyping analysis revealed that while overall gene expression did not exhibit significant shifts with treatment, certain treatments influenced the proportions of single‐cell subtypes in both PDX and PDXO models. Expansion of the majority cell subtype under SCSubtype was observed in CR models when comparing time matched pairs. Of note, this meant expansion of basal‐like proportions in all models except BCM‐7482, which saw expansion of HER2E calls. The claudin‐low subtype is not included in SCSubtype methodologies. However, our findings reveal an interesting alignment: both the CS and CR pair in the BCM‐7482 PDX and the SUM149 cell line were classified as HER2E by the majority of cell‐wise calls, despite being identified as claudin‐low in prior pseudobulk analysis. This suggests a potential overlap between the claudin‐low subtype and cell‐wise calls for the HER2E subtype under SCSubtype methodologies. Therefore, we suspect that a proportion of the cells annotated as HER2E herein may stem from the claudin‐low subtype. Also, the majority of matched CR/CS sample sets saw decrease in luminal typed cells in CR models, aligning with decreased ESR‐mediated signalling seen in pathway analysis of these models. The observed shifts towards more basal cells in many CR models suggest potential clonal selection that may be related to resistance mechanisms.

Analysis of lymph node samples revealed a trend towards lower proportions of cells aligning with the HER2E subtype compared to matched primary tissues, indicating a reduced presence of cell populations expressing genes characteristic of this subtype in metastatic lesions. These results emphasise the importance of utilising high‐resolution techniques like SCSubtype analysis to gain nuanced insight into the molecular profile of cancers. Additionally, further investigation of ER+ isogenic pairs subjected to E2‐treatment and EWD revealed pathways specifically reliant on liganded estrogen receptor, and not stimulated by constitutively active mutant ER. Understanding the distinct transcriptional programs triggered by ligand‐independent ER activity is crucial, as mutations in ER lead to constitutive activation and reduce tumour sensitivity to endocrine therapy. ESR1 mutations are prevalent in advanced and metastatic breast cancer, prompting the exploration of targeted therapeutics tailored to ESR1‐mutant tumours.^102^, ^103^ In the future, it will be essential to identify ER‐mediated transcriptional alterations unique to various mutant alleles of ESR1. This is imperative for devising precise, targeted therapeutic strategies for individuals with ESR1‐mutant conditions.

Subtype shifts in models of carboplatin resistance raised the intriguing possibility that targeted treatment may induce subtype‐specific changes, offering a glimpse into the dynamic interplay between drug response and the heterogeneity of breast cancer cell populations. This dynamic shift in subtypes might serve as an early indicator of treatment efficacy, potentially guiding more tailored therapeutic approaches. Herein we attempt to evaluate these single‐cell subtyping annotations as predictors of drug response to a number of anti‐cancer agents targeting relevant pathways. While we do not evaluate the potential of these calls to aid in sequential treatment response prediction, we feel our analysis is a necessary first step in evaluating the predictive ability of these agents.

Intriguing observations emerged regarding subtype specific responses to drug targeting, such as nuanced and varied subtype specific response to Bcl‐2 inhibitors. Furthermore, drugs that exhibited significant associations with both Luminal B and Basal subtypes in opposing directions underscore the effectiveness of SCSubtype methodologies in stratifying mixed sample sets by intrinsic subtypes and provided valuable insights into therapeutic response.

When preforming similar corollary analysis with TNBCtype subtype methods, many of the drugs correlated with LAR subtype were previously seen to be similarly associated with Luminal B subtype in our SCSubtype analysis, an unsurprising finding given the characterised similarities of these two subtypes.^15^, ^16^, ^104^, ^105^ These findings however, were particularly interesting as many drugs were seen to be negatively correlated, suggesting that while there are few LAR typed cells (< .02%) within our samples, some proportion of these cells may serve to increase the models sensitivity to agents targeting these pathways. Given the number of cells typed as LAR and the strength of response seen within these samples, we suspect there may be more LAR cells within these samples than are being called by the TNBCtype tool. Given the large viability drop seen in these LAR cell containing samples when treated with certain drugs and their divergent response, we suspect that some proportion of the untyped cells in this dataset may be LAR aligning. It is known that missingness in scRNA‐seq gene expression is not random, and is rather most often biologically or technically driven.^106^ Indeed, all samples excluded cells due to missingness for gene signatures necessary when performing the current typing methodology. We posit that TNBCtype tool may under call LAR subtype within these samples due to the divergent gene expression of LAR aligning cells, excluding many of these cells from inclusion in this typing analysis.

Of note, only TNBC PDXO and cell lines are represented here, and no HER2‐amplified PDX models were included in this dataset, limiting the conclusions to be drawn from these models specifically. We further acknowledge the constraints of this study, as our sample set showed limited variety in SCSubtype and TNBCtype subtyped samples. These limitations represent areas for further study of model and subtype differences which might serve to advance our understanding of breast malignancies.

## 4 CONCLUSIONS

Herein we present a detailed transcriptional atlas of human breast cancer cells. This comprehensive dataset incorporates various sample types, treatments, and isogenic pairs of drug‐resistant/sensitive models. While presenting this integration of cells, we have demonstrated the utility of dynamic subtyping strategies in predicting therapeutic treatment responses, thus paving the way for more personalised and effective breast cancer treatment approaches. Furthermore, our study elucidates subtype‐specific and model‐specific insights. These novel findings related to metabolic profiles and subtype shifts underscore the complexity of breast cancer, urging continued efforts to refine and advance our understanding for improved clinical decision‐making and model development.

## 5 MATERIALS AND METHODS

### 5.1 TNBC cell line culture

Cells were cultured in RPMI‐1640 GlutaMAX media (ThermoFisher Scientific) supplemented with penicillin, streptomycin, and 10% fetal bovine serum. Previously published protocols and information on where cell lines were obtained can be found at https://doi.org/10.1016/j.tranon.2021.101235.^107^

### 5.2 PDX culture

HCI‐001, HCI‐011, and HCI‐013 were obtained from the Huntsman Cancer Institute. BCM‐0132, BCM‐2147, BCM‐2277, BCM‐3887, BCM‐5097, BCM‐7482, BCM‐7821, BCM‐15034, and BCM‐15057 were obtained from the Baylor College of Medicine. UCD52 was obtained from the University of Colorado, Denver. WHIM2 and WHIM30 were obtained from Washington University in St. Louis. VCU‐BC‐001, VCU‐BC‐002, VCU‐BC‐003, VCU‐BC‐004, VCU‐BC‐006, and VCU‐BC‐024 were obtained from the Virginia Commonwealth University Mouse Models Core. The Institutional Animal Care and Use Committee (IACUC) at Virginia Commonwealth University (VCU) gave its approval for studies involving mice (Protocol# AD10001247; approved June 29, 2018), and all experiments were carried out in compliance with IACUC rules and regulations. Non‐obese diabetic severe mixed immunodeficient gamma (NSG) mice were used for this study. The NSG mice were bred by VCU Cancer Mouse Models Core. Tumour cells were resuspended in Matrigel (Corning) and injected into the 4th mammary fat pads. Tumours were collected at ∼10 × 10 and were digested in DMEM/F12, 5% fetal bovine serum (FBS), 300 U/mL collagenase (Sigma), and 100 U/mL hyaluronidase solutions (Sigma).^108^ Digested tumours were trypsinized and single cells were resuspended in a .04% BSA, PBS solution prior to single‐cell collection.

### 5.3 PDXO culture

To prepare PDXOs, tumours were resected from PDX models and finely chopped using a sterile razor blade and placed into a solution for tumour digestion (DMEM/F12 containing 5% FBS, .0533 mg/mL hyaluronidase, and 2.4 mg/mL collagenase) in a temperature‐controlled tube cycler set at 37°C for 1 h. Following this, the solutions were centrifuged, and the resulting pellets were treated with red blood cell lysis buffer, centrifuged again, and the supernatants were discarded. A single‐cell suspension was obtained by subjecting the cells to trypsin digestion. Subsequently, the cells were suspended in Hanks’ balanced salt solution (HBSS) supplemented with 2% FBS for subsequent procedures. Depletion of mouse cells was carried out using Miltenyi Biotec Mouse Cell Depletion Kit according to kit specifications.^109^ Following mouse cell depletion, cells were then embedded in 150‐μL Cultrex domes and plated onto a 50‐μL Cultrex base layer in six‐well tissue culture plates. The plates were inverted and briefly incubated to solidify the Cultrex domes, after which culture medium was added. This medium consisted of Advanced DMEM/F12 with 5% FBS, 10 mM HEPES, 1× Glutamax, 1 μg/mL hydrocortisone, 50 μg/mL gentamicin, and 10 ng/mL hEGF, supplemented with 10 μM Y‐27632. Medium exchange occurred every 3 to 4 days, and once mature, cultures were passaged using dispase solution followed by a dissociation step in TrypLE Express. For passaging, single cells were seeded at a density of 200 000–400 000 cells per dome. For single‐cell collection, trypsinized single cells were resuspended in a .04% BSA following dissociation steps in TrypLE. PDXO protocol was amended from Guillen et al.^36^

### 5.4 10X Genomics Chromium Next GEM library construction and sequencing

With regards to PDX, PDXO and cell line data, the Chromium Single Cell 3′ Protocol was followed according to manufacturer‐recommendations for single‐cell captures and cDNA preparations using the 10X Genomics Chromium machine. The GEM reaction mixture was cleaned with Dynabeads MyOne SILANE (10X Genomics PN#2000048) before the barcoded cDNA was amplified in a subsequent PCR process. Following this, a secondary clean‐up step was performed using SPRIselect reagent (Beckman Coulter #B23318). Agilent Bioanalyzer High Sensitivity chip was used to quality control check cDNA for each sample prior to library preparation. Gene expression library construction was done according to 10X Chromium specification. Agilent Bioanalyzer High Sensitivity chip was used to quality control library preparations prior to sequencing. Methods for library construction and sequencing of human primary patient samples can be found at https://doi.org/10.15252/embj.2020107333.26 Single‐cell RNA sequencing (scRNA‐Seq) was performed on 4 cell line, 42 PDX, and 2 PDXO samples using Illumina NextSeq 2000 or Illumina HiSeq 4000 platforms. Collection of 69 primary patient samples and sequencing was performed as previously described.^26^, ^110^ PDX, PDXO and cell line samples were sequenced in the VCU Genomics core facilities. Primary patient samples were collected and sequenced by the Walter and Eliza Hall Institute (WEHI) in Melbourne, Australia. All raw and processed data from VCU and processed data from WEHI have been uploaded to the Gene Expression Omnibus under accession GSE276609.

### 5.5 Single‐cell RNA‐seq bioinformatic analysis

#### 5.5.1 Sample‐level QC and alignment

Raw Fastq files were first assessed for quality using FastQC v0.11.9^111^ and MultiQC v1.11^112^ prior to further analysis. Alignment of all samples was performed using the 10X Genomics CellRanger v6.0.1 ‘count’ algorithm. PDX samples were first aligned to the 10X Genomics generated GRCh38/mm10 multispecies genome. The secondary analysis ‘gem_classification.csv’ file, which makes calls for human, mouse, and multiplet cells,^113^ was utilised to differentiate mouse and human cells and remove multiple cells. Barcodes associated with human cells were extracted from the aligned BAM files and converted back to FASTQ format for re‐alignment to the 10X Genomics GRCh38 human genome. The cell line, PDXO, and patient samples were all aligned directly to the 10X Genomics GRCh38 human genome. From this point all samples were run through the same pipeline using the human data only.

#### 5.5.2 Cell‐level QC

Dying and multiplet human cells were removed from the dataset using R v4.1.3, the Seurat v.4.3.0 package^114^ and an internal R script. Briefly, poor quality cells were identified using mitochondrial gene expression, number of detected genes, and unique molecular index (UMI) counts using 10X Genomic guidelines.^115^ Filtering thresholds for each metric were determined individually for each sample by using 3 median absolute deviations (MAD) above the mean for mitochondrial expression, and above and below for gene and UMI counts. Cells not meeting these thresholds were removed from further analysis due to poor quality. Of note, the mitochondrial MAD threshold was calculated using only cells with less than 50% of mitochondrial expression due to several samples having high mitochondrial content. Additionally, the maximum allowed per cent mitochondrial gene expression for any cell was capped at 25% regardless of the median for that sample, and any sample with a median less than 5% was automatically increased to 25%. These exceptions were implemented to ensure samples had a reasonable number of cells returned with less than 25% mitochondrial expression. Information on read count, cell count, and mitochondrial expression cutoffs can be found in Supplemental File 1.

#### 5.5.3 Sample merging and Loupe file generation

Following removal of poor‐quality cells, samples were merged using an in‐house R script that utilises Seurat's ‘merge()’ function. Briefly, the 10X Genomics filtered feature barcode data was imported into R using the Seurat package. Barcodes were edited to have a consecutively increasing postfix number to differentiate samples. Prior to merging, we minimised the impact of technical variability across samples through applying Seurat's ‘NormalizeData()’ and ‘ScaleData()’ functions on each sample before merging. Normalisation was achieved with Log2 Normalisation, which has been found to perform as well or better than more sophisticated transformation methods,^116^ so that variance differences across samples are minimised. This was followed by finding variable features with ‘FindVariableFeatures()’ and scaling the data so gene expression values are between 0 and 1 with the ‘ScaleData()’ function following the standard Seurat pipeline as described in Dave et al and elsewhere in the literature.^117^ To avoid introducing computational artefacts, batch correction methods were not utilised to combine data as many of these methods assume samples contain the same populations of cells,^118^ which is a poor fit for this dataset. It is recommended that any smaller subset of our data, where all samples can be assumed to contain the same cell populations, be integrated using Harmony prior to analysis (e.g. isogenic samples). Merging was then performed iteratively using Seurat's ‘merge()’ function and a list of Seurat objects. Once merged, the tSNE and UMAP visualisations, along with cell clustering analysis, were generated followed by a cell cycle analysis using Seurat's ‘CellCycleScoring()’ function to obtain the cell cycle phase for each cell. At this point, additional sample‐level annotations were mapped to individual cells, such as treatment, PDX type, and source tissue. UMAP, tSNE, clustering results, cell cycle and other annotations were saved to Loupe‐compatible comma separated value (CSV) files. The raw read count matrix was then saved as a 10X Genomics formatted H5 file using the ‘write10xCounts’ function from the DropletUtils R package, which was then processed by the CellRanger ‘reanalyze’ algorithm to produce a Loupe file for visualisation and downstream analyses. The CSV formatted annotations and visualisations were manually imported into the generated Loupe file. Since the first submission of this paper 10X Genomics has released the RLoupe package to convert Seurat objects directly to a .cloupe file format. The data presented here was gathered prior to this package release and was converted to a .cloupe file using the method above.

### 5.6 Differentially expressed gene (DEG) and pathway analysis

Differentially expressed gene (DEG) analysis was performed in 10X Genomics Loupe Browser v.7.0.0, using the ‘Run Differential Expression’ function with ‘Between selected cluster(s) themselves’ selected.

#### 5.6.1 CS/CR analysis

In the instances of the UCD52, WHIM30 and BCM‐2147 PDX models, as well as the WHIM30 PDXO model, time‐matched samples were collected. These samples underwent library preparation and sequencing in the same batch, thereby mitigating potential batch effects and enhancing the comparative potential of these models. For additional model BCM‐7482 we sequenced single‐cell libraries for the developed CR models without a time matched pair and performed comparison with their previously sequenced CS pair following normalisation steps to reduce batch effect.

DEG was performed in five batches for CS/CR comparisons, utilising time matched samples where available:

(1) BCM‐2147_109176/BCM‐2147CR_109178
(2) BCM‐7482_107157/BCM‐7482CR_109078
(3) UCD52_107080‐1/UCD52CR_107086 & UCD52CR_107269
(4) WHIM30_108896/WHIM30CR_108903
(5) WHIM30CR‐O_108805‐9/WHIM30‐O_108793‐4

DEG batches were exported from loupe browser into .csv file format with adjusted *p*‐values, log2 fold change, and median expression values for all genes which were not annotated as having ‘low average count’ (defined by an average occurrence less than 1 count per cell across the dataset).

Gene expression data was imported into Qiagen Ingenuity Pathway Analysis (IPA) software.^119^ A core analysis was created for each set of samples, with settings ‘Expression Analysis’ and ‘Expr Log Ratio’ in IPA with thresholds for feature inclusion of *p*‐value < .1 and log2 fold change cutoffs of < –.6 or > .6. Following core analyses, a comparative analysis was created for all groups, IPA z‐scores were analysed for trends specific to differences between CR/CS pairs.

#### 5.6.2 E2‐treated/EWD/EI analysis

DEG was performed in 3 batches for estradiol withdrawal comparisons, utilising time matched samples where available:

(1) BCM‐15057_107673/ BCM‐15057_107684
(2) HCI‐011_107334/HCI‐011_107332
(3) HCI‐013_10666‐10667/HCI‐013_106662 & HCI‐013_106663

Similarly to the above CR/CS analysis, batches were exported from loupe browser into .csv file format with adjusted *p*‐values, log2 fold change, and median expression values for all genes which were not annotated as having ‘low average count’ (defined by an average occurrence less than 1 count per cell across the dataset).

Gene expression data was imported into Qiagen Ingenuity Pathway Analysis (IPA) software.^119^ A core analysis was created for each set of samples, with settings ‘Expression Analysis’ and ‘Expr Log Ratio’ in IPA with thresholds for feature inclusion of *p*‐value < .1 and log2 fold change cutoffs of <–.6 or >.6. Following core analyses, a comparative analysis was created for all groups, IPA z‐scores were analysed for trends specific to differences between E2‐treated and EWD/EI pairs.

### 5.7 InferCNV analysis

Inference of single‐cell copy number variations were done utilising the R packages scTyper^120^ and inferCNV v1.3.3.^121^  Custom functions and some code from the scTyper R package^122^ was modified for use independently of scTyper and utilised in out in‐house script as we had difficulty getting scTyper to run successfully.  Our in‐house inferCNV analysis script includes customisations to run inferCNV over our large breast cancer dataset in batches due to the high computational resources required, as well as generating Z‐scores of the calculated inferCNV score so that batch results could be directly compared. 

Prior to running inferCNV, the merged dataset that contains tumour, normal, and immune cells was subset to 30% of the cells in each sample as the full dataset was too large to process all at once. InferCNV was run in batches of 10 samples at a time.  Five of those were randomly chosen normal samples that were used as the reference group in each run of inferCNV so that all samples were compared to the same set of normal data: N‐0019‐total, N‐0021‐total, N‐0064‐total, N‐0092‐total, N‐0093‐total.  The other five samples ran in batches.  Output included an inferCNV score for each cell. The inferCNV score mean and standard deviation of the reference group was calculated and used to generate Z‐scores for the entire cohort.  Thus, the reference and other normal samples should have low z‐scores around zero, and significant deviations from zero should indicate malignant cells. Scores were discretised and added as an annotation to the Loupe file for analysis. Z‐scores ranged from –2 to 32.

### 5.8 Malignant cell identification and cancer‐only datasets

InferCNV was utilised to obtain cell‐level CNV scores to identify malignant versus normal cells in a subset of cells from the ‘Master Merge’ (see Supplementary Methods for details). An in‐house R script calculated the mean and standard deviation of the inferCNV scores for the reference set of samples that was then used to calculate a Z‐Score for the rest of the cells in the dataset. Thus, reference and other normal samples will have a z‐score around zero, and significant deviations from zero will indicate cancer cells. The z‐score data was discretised and uploaded into the Loupe Browser for the ‘Master Merge’. We manually identified consistent patterns where low z‐score cells from PDX, or patient samples clustered near or in normal cell clusters. Recognising this strong pattern and the computationally expensive pipeline of running InferCNV on the rest of the dataset, we chose to forgo InferCNV processing in favour of identifying and selecting all cells clustering with normal samples in the ‘Master Merge’ as non‐malignant cells that needed to be removed from the dataset moving forward.

Utilising the ‘Master Merge’ Loupe file three main groups of cells were manually annotated in the Loupe Browser: immune, normal, and malignant cells. Normal cells included all normal samples as well as any PDX or patient cells that clustered with the normal cells based on the UMAP visualisation. Those barcodes annotated as ‘malignant’ were exported to a TSV file with all headers and annotation columns removed. An in‐house R script^123^ extracted each set of barcodes for each sample, replaced the numbered postfix on the barcode from the merged dataset to ‘–1’, and saved a new TSV file in the individual sample's results directory. The in‐house script also re‐generated the config file for the in‐house merge script by replacing any previous barcode list of cells to keep with the newly generated cancer cell‐only file. This config script was then utilised in another merge run to create the cancer‐only dataset.

The cancer‐only dataset contains 89 samples with a total of 260 500 cells. This dataset was further divided into TNBC‐ and ER+‐only subsets where the TNBC‐only dataset contains 40 samples with 111 442 cells and the ER‐only subset contains 42 samples with 131 471 cells.

### 5.9 Subtype analysis

#### 5.9.1 Cancer‐only dataset

Subtyping the cancer‐only dataset was performed using two methods, pseudo‐bulk and SCSubtype. The pseudo‐bulk method, implemented with an in‐house R script, was used to classify samples into PAM50 subtypes (Basal, Her2, LumA, LumB, Normal‐like) and identify which samples were also Claudin‐low. The SCSubtype method is the intrinsic single‐cell subtyping method developed by Wu et al. that performs subtyping at the cell level to classify individual cells into one of four PAM50 subtypes (Basal‐like, Her2E, LumA, LumB).^10^

The pseudo‐bulk PAM50 subtyping was performed by first acquiring the pseudo‐bulk gene expression for each sample through summing all read counts for each gene across all cells in a sample with Seurat's ‘AggregateExpression()’ function. Now instead of having hundreds or thousands of expression values per gene in a sample (one for each cell) we have one aggregated value per gene per sample. This data was normalised using the DESeq2 v1.42 normalised counts median of ratio's method.^124^ The gene list was then filtered to those that had an Entrez identifier and gene symbol prior to running subtyping using the Genefu v2.34 ‘molecular.subtyping’ function with ‘sbt.model’ set to ‘pam50’. Claudin‐low classifications were obtained by utilising the same pseudo‐bulk counts with the ‘claudinLow()’ function available through Genefu.^39^, ^47^

Code for SCSubtype was obtained from the Swarbricklab‐code GitHub repository^125^ on 6/28/2023. Two files were downloaded: Highest_calls.R and NatGen_Supplementary_table_S4.csv. The R file was modified so that it could be run from the command line, output status messages, and reformat the output files to be Loupe‐compatible–no other code was altered. The merged cancer‐only dataset, formatted as a Seurat object, and the downloaded NatGen_Supplementary_table_S4.csv file, which contained the PAM50 gene signatures, were used as inputs to the SCSubtype script. Output annotations were then manually imported into the cancer‐only Loupe file for analysis.

#### 5.9.2 TNBC‐only dataset

A subset of the cancer‐only dataset was generated to consist of TNBC samples only. These samples were then further characterised by utilising the TNBCtype subtyping method developed by Chen et al.^126^ Briefly, The TNBCtype software was used to classify each candidate TNBC sample into four TNBC subtypes using centroid correlation to the BL1, BL2, LAR, and M subtypes using the highest positive centroid correlation. TNBC subtyping was performed on normalised expression from each individual epithelial cell. Cells that did not have the required depth of expression for the genes in the TNBC signature were excluded from the analysis and labelled as ‘None’ in Figure 9.

### 5.10 Statistical analysis of drug screening

As previously reported, high‐throughput screening was carried out on PDX models of ER+ and TNBC cancers utilising a 555‐drug library at 1 and 10 μM concentration.^50^, ^61^ TNBCtype and SCSubtype were applied at the single‐cell level was applied to relevant PDX models to estimate the proportion of subtype‐aligning cells within each PDX model (as described in above). Once subtype proportions were determined for relevant PDX models, those subtyped proportions could be integrated with drug screening information. Drug screening data for these 555 anti‐cancer compounds were then integrated with projected proportions of cells categorised by various PDX models.

This analysis involved hierarchical clustering of the 24 included PDX models based on their projected TNBCtype proportions, resulting in four main clusters primarily driven by proportions of M and BL1 subtypes.

Correlation analysis, using the cor() function in R with a suggestive threshold of *p* < .1 and significance threshold of *p* < .05 following BH multiple testing correction, explored associations between TNBCtype subtypes and drug responses at concentration of 1 μM.

Expanding the analysis to clinically typed TNBC and ER+ PDX models, significant associations between SCSubtype proportions and cellular viability post‐pharmacological intervention were similarly observed through correlation analysis.

## AUTHOR CONTRIBUTIONS

Julia E. Altman and J. Chuck Harrell conceived the project and directed the study with input from all authors. Julia E. Altman, Amy L. Olex, and J. Chuck Harrell contributed to the experimental design. Julia E. Altman, Amy L. Olex, Emily K. Zboril, Mikhail G. Dozmorov, X. Steven Chen, Charles M. Perou, Brian D. Lehmann, Jane E. Visvader, and J. Chuck Harrell assisted with interpreting the data. Julia E. Altman, Amy L. Olex, X. Steven Chen, and Yunshun Chen performed bioinformatic analyses. Julia E. Altman, Emily K. Zboril, Carson J. Walker, David C. Boyd, Rachel K. Myrick, Nicole S. Hairr, Jennifer E. Koblinski, Madhavi Puchalapalli, and Bin Hu collected and provided PDX tissues. Julia E. Altman, Emily K. Zboril, Carson J. Walker, David C. Boyd, Rachel K. Myrick, and Nicole S. Hairr performed the tumour dissociation experiments. Julia E. Altman performed the single‐cell captures, scRNA‐seq experiments on the Chromium Controller, and prepared the next‐generation sequencing of the scRNA‐seq libraries. Julia E. Altman and Amy L. Olex performed the preprocessing for the scRNA‐seq data. Amy L. Olex performed data integration and clustering of scRNA‐seq data. Julia E. Altman performed gene signature analyses and identification of stromal, epithelial, and normal cells, with intellectual input from J. Chuck Harrell and Charles M. Perou. Amy L. Olex performed the analysis and benchmarking of inferCNV, application of SCSubtype, and PAM50 centroid predictor. X. Steven Chen applied TNBCtype to single cells. Julia E. Altman performed DEG/Pathway analysis. Mikhail G. Dozmorov performed statistical analyses. Julia E. Altman, Emily K. Zboril, Carson J. Walker, and David C. Boyd preformed high‐throughput drug screening on PDX tissues. Julia E. Altman performed correlation analysis with subtype projections. Julia E. Altman designed and generated figures with intellectual input from Emily K. Zboril, Brian D. Lehmann and J. Chuck Harrell. Julia E. Altman wrote the manuscript with input from all authors.

## CONFLICT OF INTEREST STATEMENT

The following authors disclosed conflicts of interest. Charles M. Perou is listed as an inventor on patent applications on the Breast PAM50 assay and is an equity stock holder and consultant of BioClassifier LLC. BDL and XSC are inventors (US Patent No. 11788147) of intellectual property (TNBCtype) licensed by Oncocyte Corp; the licensed IP is indirectly related to the work.

## ETHICS STATEMENT

All research within this manuscript followed ethical guidelines aligning with the National Institute of Health and Virginia Commonwealth University ethics trainings. Breast tissues from consenting patients, including normal tissue, tumours, and lymph nodes, were acquired through the Royal Melbourne Hospital Tissue Bank, the Victorian Cancer Biobank, and kConFab, with approval from the relevant institutional review boards. Human Ethics approval was granted by the Walter and Eliza Hall Institute (WEHI) Human Research Ethics Committee. Only de‐identified human material was analysed for this study. All research involving animals was conducted in accordance with a protocol approved by the institutional animal care and use committee (IACUC [Protocol #AD10001247]).

## Supporting information



Supporting information

Supporting information

Supporting information

Supporting information

## Data Availability

The datasets supporting the conclusions of this article are available in The Gene Expression Omnibus. Processed scRNA‐seq data and Loupe Browser files from this study may be found under accession number GSE276609. Bioinformatic code and in‐house scripts are provided on GitHub (https://github.com/VCUWrightCenter/decoding‐breast‐cancer).

## References

[1] Siegel RL , Miller KD , Wagle NS , Jemal A . Cancer statistics, 2023. Cancer J Clin. 2023;73:17‐48. 10.3322/caac.21763 36633525

[2] Desantis CE , Ma J , Gaudet MM . Breast cancer statistics, 2019. Cancer J Clin. 2019;69:438‐451. 10.3322/caac.21583 31577379

[3] Ma J , Jemal A . Breast Cancer Metastasis and Drug Resistance: Progress and Prospects. Springer; 2013:1‐18. doi:10.1007/978-1-4614-5647-6_1. Breast Cancer Statistics. ed. Ahmad, A..

[4] The Cancer Genome Atlas Network . Comprehensive molecular portraits of human breast tumours. Nature. 2012;490:61‐70. 23000897 10.1038/nature11412 PMC3465532

[5] Kandoth C , Mclellan MD , Vandin F , et al. Mutational landscape and significance across 12 major cancer types. Nature. 2013;502:333‐339. 24132290 10.1038/nature12634 PMC3927368

[6] Curtis C , Shah SP , Chin S‐F , et al. The genomic and transcriptomic architecture of 2,000 breast tumours reveals novel subgroups. Nature. 2012;486:346‐352. 22522925 10.1038/nature10983 PMC3440846

[7] Huber KE , Carey LA , Wazer DE . Breast cancer molecular subtypes in patients with locally advanced disease: impact on prognosis, patterns of recurrence, and response to therapy. Semin Radiat Oncol. 2009;19(4):204‐210. 19732684 10.1016/j.semradonc.2009.05.004

[8] Perou CM , Sørlie T , Eisen MB , et al. Molecular portraits of human breast tumours. Nature. 2000;406:747‐752. 10963602 10.1038/35021093

[9] Sørlie T , Perou CM , Tibshirani R , et al. Gene expression patterns of breast carcinomas distinguish tumor subclasses with clinical implications. Proc Natl Acad Sci. 2001;98:10869‐10874. 11553815 10.1073/pnas.191367098 PMC58566

[10] Wu SZ , Al‐Eryani G , Roden DL , et al. A single‐cell and spatially resolved atlas of human breast cancers. Nat Genet. 2021;53:1334‐1347. 34493872 10.1038/s41588-021-00911-1 PMC9044823

[11] Sørlie T , Tibshirani R , Parker J , et al. Repeated observation of breast tumor subtypes in independent gene expression data sets. Proc Natl Acad Sci. 2003;100:8418‐8423. 12829800 10.1073/pnas.0932692100 PMC166244

[12] Testa U , Castelli G , Pelosi E . Breast cancer: a molecularly heterogenous disease needing subtype‐specific treatments. Med Sci. 2020;8:18. 10.3390/medsci8010018 PMC7151639 32210163

[13] Myers MB . Targeted therapies with companion diagnostics in the management of breast cancer: current perspectives. Pharmacogenomics Pers Med. 2016:7‐16. 10.2147/PGPM.S56055 PMC4730993 26858530

[14] Essadi I , Benbrahim Z , Kaakoua M , Reverdy T , Corbaux P , Freyer G . HER2‐positive metastatic breast cancer: available treatments and current developments. Cancers. 2023;15:1738. 36980624 10.3390/cancers15061738 PMC10046228

[15] Lehmann BD , Bauer JA , Chen Xi , et al. Identification of human triple‐negative breast cancer subtypes and preclinical models for selection of targeted therapies. J Clin Invest. 2011;121:2750‐2767. 21633166 10.1172/JCI45014 PMC3127435

[16] Lehmann BD , Jovanović B , Chen Xi , et al. Refinement of triple‐negative breast cancer molecular subtypes: implications for neoadjuvant chemotherapy selection. PLOS ONE. 2016;11:e0157368. 27310713 10.1371/journal.pone.0157368 PMC4911051

[17] Ismail‐Khan R , Bui MM . A review of triple‐negative breast cancer. Cancer Control. 2010;17:173‐176. 20664514 10.1177/107327481001700305

[18] Dent R , Trudeau M , Pritchard KI , et al. Triple‐negative breast cancer: clinical features and patterns of recurrence. Clin Cancer Res. 2007;13:4429‐4434. 17671126 10.1158/1078-0432.CCR-06-3045

[19] Qiu J , Xue X , Hu C , et al. Comparison of clinicopathological features and prognosis in triple‐negative and non‐triple negative breast cancer. J Cancer. 2016;7:167. 26819640 10.7150/jca.10944 PMC4716849

[20] Yuan ZY , et al. Clinical characteristics and prognosis of triple‐negative breast cancer: a report of 305 cases. Ai Zheng Aizheng Chin J Cancer. 2008;27:561‐565. 18570725

[21] Shapiro E , Biezuner T , Linnarsson S . Single‐cell sequencing‐based technologies will revolutionize whole‐organism science. Nat Rev Genet. 2013;14:618‐630. 23897237 10.1038/nrg3542

[22] Sharma SV , Lee DY , Li B , et al. A chromatin‐mediated reversible drug‐tolerant state in cancer cell subpopulations. Cell. 2010;141:69‐80. 20371346 10.1016/j.cell.2010.02.027 PMC2851638

[23] Rosati D , Giordano A . Single‐cell RNA sequencing and bioinformatics as tools to decipher cancer heterogenicity and mechanisms of drug resistance. Biochem Pharmacol. 2022;195:114811. 34673017 10.1016/j.bcp.2021.114811

[24] Lee HW , et al. Single‐cell RNA sequencing reveals the tumor microenvironment and facilitates strategic choices to circumvent treatment failure in a chemorefractory bladder cancer patient. Genome Med. 2022;12:1‐21. 10.1186/s13073-020-00741-6 PMC7251908 32460812

[25] Kan T , Zhang S , Zhou S , et al. Single‐cell RNA‐seq recognized the initiator of epithelial ovarian cancer recurrence. Oncogene. 2022;41:895‐906. 34992217 10.1038/s41388-021-02139-z

[26] B P , et al. A single‐cell RNA expression atlas of normal, preneoplastic and tumorigenic states in the human breast. EMBO J. 2021;40. 10.15252/embj.2020107333 PMC8167363 33950524

[27] Rakha EA , El‐Sayed ME , Green AR , Lee AHS , Robertson JF , Ellis IO . Prognostic markers in triple‐negative breast cancer. Cancer. 2007;109:25‐32. 17146782 10.1002/cncr.22381

[28] Imrich S , Hachmeister M , Gires O . EpCAM and its potential role in tumor‐initiating cells. Cell Adhes Migr. 2012;6:30‐38. 10.4161/cam.18953 PMC3364135 22647938

[29] Ring A , Mineyev N , Zhu W , et al. EpCAM based capture detects and recovers circulating tumor cells from all subtypes of breast cancer except claudin‐low. Oncotarget. 2015;6:44623. 26556851 10.18632/oncotarget.5977 PMC4792580

[30] Lim E , Wu Di , Pal B , et al. Transcriptome analyses of mouse and human mammary cell subpopulations reveal multiple conserved genes and pathways. Breast Cancer Res. 2010;12:1‐14. 10.1186/bcr2560 PMC2879567 20346151

[31] Sebastian A , Hum NR , Martin KA , et al. Single‐cell transcriptomic analysis of tumor‐derived fibroblasts and normal tissue‐resident fibroblasts reveals fibroblast heterogeneity in breast cancer. Cancers. 2020;12:1307. 32455670 10.3390/cancers12051307 PMC7281266

[32] García‐Teijido P , Cabal ML , Fernández IP , Pérez YF . Tumor‐infiltrating lymphocytes in triple negative breast cancer: the future of immune targeting. Clin Med Insights Oncol. 2016;10s1:CMO.S34540. 10.4137/CMO.S34540 PMC4822722 27081325

[33] Vonderheide RH , Domchek SM , Clark AS . Immunotherapy for breast cancer: what are we missing. Clin Cancer Res Off J Am Assoc Cancer Res. 2017;23:2640‐2646. 10.1158/1078-0432.CCR-16-2569 PMC5480967 28572258

[34] Goldberg J , Pastorello RG , Vallius T , et al. The immunology of hormone receptor positive breast cancer. Front Immunol. 2021;12:674192. 34135901 10.3389/fimmu.2021.674192 PMC8202289

[35] Go RS , Adjei AA . Review of the comparative pharmacology and clinical activity of cisplatin and carboplatin. J Clin Oncol. 1999;17:409‐409. 10458260 10.1200/JCO.1999.17.1.409

[36] Guillen KP , Fujita M , Butterfield AJ , et al. A human breast cancer‐derived xenograft and organoid platform for drug discovery and precision oncology. Nat Cancer. 2022;3:232‐250. 35221336 10.1038/s43018-022-00337-6 PMC8882468

[37] Kang YP , Mockabee‐Macias A , Jiang C , et al. Non‐canonical glutamate‐cysteine ligase activity protects against ferroptosis. Cell Metab. 2021;33:174‐189. 33357455 10.1016/j.cmet.2020.12.007 PMC7839835

[38] Parker JS , Mullins M , Cheang MCU , et al. Supervised risk predictor of breast cancer based on intrinsic subtypes. J Clin Oncol. 2009;27:1160. 19204204 10.1200/JCO.2008.18.1370 PMC2667820

[39] Gendoo DMA , Ratanasirigulchai N , Schroeder MS , et al. genefu: Computation of Gene Expression‐Based Signatures in Breast Cancer. [Internet]. 2020. Available from: https://bioconductor.org/packages/genefu 10.1093/bioinformatics/btv693 PMC6410906 26607490

[40] Beltjens F , Molly D , Bertaut A , et al. ER−/PR+ breast cancer: a distinct entity, which is morphologically and molecularly close to triple‐negative breast cancer. Int J Cancer. 2021;149:200‐213. 33634878 10.1002/ijc.33539

[41] Groenendijk FH , Treece T , Yoder E , et al. Estrogen receptor variants in ER‐positive basal‐type breast cancers responding to therapy like ER‐negative breast cancers. Npj Breast Cancer. 2019;5:1‐8. 31016233 10.1038/s41523-019-0109-7 PMC6472385

[42] Iwamoto T , Booser D , Valero V , et al. Estrogen receptor (ER) mRNA and ER‐related gene expression in breast cancers that are 1% to 10% ER‐positive by immunohistochemistry. J Clin Oncol. 2012;30:729‐734. 22291085 10.1200/JCO.2011.36.2574

[43] Deyarmin B , Kane JL , Valente AL , et al. Effect of ASCO/CAP guidelines for determining ER status on molecular subtype. Ann Surg Oncol. 2013;20:87‐93. 22875649 10.1245/s10434-012-2588-8

[44] Thompson KJ , Leon‐Ferre RA , Sinnwell JP , et al. Luminal androgen receptor breast cancer subtype and investigation of the microenvironment and neoadjuvant chemotherapy response. NAR Cancer. 2022;4:zcac018. 35734391 10.1093/narcan/zcac018 PMC9204893

[45] Lehmann BD , Bauer JA , Schafer JM , et al. PIK3CA mutations in androgen receptor‐positive triple negative breast cancer confer sensitivity to the combination of PI3K and androgen receptor inhibitors. Breast Cancer Res. 2014;16:1‐14. 10.1186/s13058-014-0406-x PMC4187324 25103565

[46] Voutsadakis IA . Comparison of clinical subtypes of breast cancer within the claudin‐low molecular cluster reveals distinct phenotypes. Cancers. 2023;15:2689. 37345027 10.3390/cancers15102689 PMC10216296

[47] Prat A , Parker JS , Karginova O , et al. Phenotypic and molecular characterization of the claudin‐low intrinsic subtype of breast cancer. Breast Cancer Res. 2010;12:R68. 20813035 10.1186/bcr2635 PMC3096954

[48] Prat A , Karginova O , Parker JS , et al. Characterization of cell lines derived from breast cancers and normal mammary tissues for the study of the intrinsic molecular subtypes. Breast Cancer Res Treat. 2013;142:237‐255. 24162158 10.1007/s10549-013-2743-3 PMC3832776

[49] Thennavan A . Adapting bulk and single cell RNA sequencing for molecular analysis of breast pathology. The University of North Carolina at Chapel Hill, United States – North Carolina; 2022.

[50] Zboril EK , Grible JM , Boyd DC , et al. Stratification of tamoxifen synergistic combinations for the treatment of ER+ breast cancer. Cancers. 2023;15:3179. 37370789 10.3390/cancers15123179 PMC10296623

[51] Tzukerman M , et al. The human estrogen receptor has transcriptional activator and repressor functions in the absence of ligand. New Biol. 1990;2:613‐620. 2083252

[52] Shang Y , Hu X , DiRenzo J , Lazar MA , Brown M . Cofactor dynamics and sufficiency in estrogen receptor–regulated transcription. Cell. 2000;103:843‐852. 11136970 10.1016/s0092-8674(00)00188-4

[53] Kim MY,. A role for coactivators and histone acetylation in estrogen receptor α‐mediated transcription initiation. EMBO J. 2001;20:6084‐6094. 11689448 10.1093/emboj/20.21.6084 PMC125694

[54] Cho H , Katzenellenbogen BS . Synergistic activation of estrogen receptor‐mediated transcription by estradiol and protein kinase activators. Mol Endocrinol. 1993;7:441‐452. 7683375 10.1210/mend.7.3.7683375

[55] Gronemeyer H . Transcription activation by estrogen and progesterone receptors. Annu Rev Genet. 1991;25:89‐123. 1667464 10.1146/annurev.ge.25.120191.000513

[56] Jones PS , Parrott E , White INH . Activation of transcription by estrogen receptor α and β is cell type‐ and promoter‐dependent. J Biol Chem. 1999;274:32008‐32014. 10542232 10.1074/jbc.274.45.32008

[57] Hashimoto Y , Masunaga N , Kagara N , et al. Detection of ultra‐rare ESR1 mutations in primary breast cancer using LNA‐Clamp ddPCR. Cancers. 2023;15:2632. 37174098 10.3390/cancers15092632 PMC10177270

[58] Jeselsohn R , Bergholz JS , Pun M , et al. Allele‐specific chromatin recruitment and therapeutic vulnerabilities of ESR1 activating mutations. Cancer Cell. 2018;33:173‐186. 29438694 10.1016/j.ccell.2018.01.004 PMC5813700

[59] Vtorushin S , Dulesova A , Krakhmal N . Luminal androgen receptor (LAR) subtype of triple‐negative breast cancer: molecular, morphological, and clinical features. J Zhejiang Univ Sci B. 2022;23:617‐624. 35953756 10.1631/jzus.B2200113 PMC9381326

[60] The NExT Screening Libraries (Pre‐plated Copies Available) | Discovery | NExT Resources | NExT. Accessed January 4, 2024. https://next.cancer.gov/discoveryresources/resources_ndl.htm#oncology_interrogation_tools

[61] Turner TH , Alzubi MA , Harrell JC . Identification of synergistic drug combinations using breast cancer patient‐derived xenografts. Sci Rep. 2020;10:1493. 32001757 10.1038/s41598-020-58438-0 PMC6992640

[62] Boyd DC , Zboril EK , Olex AL , et al. Discovering synergistic compounds with BYL‐719 in PI3K overactivated basal‐like PDXs. Cancers. 2023;15:1582. 36900375 10.3390/cancers15051582 PMC10001201

[63] Budimir I , Tomasović‐Lončarić Č , Kralik K , et al. Higher expressions of SHH and AR are associated with a positive receptor status and have impact on survival in a cohort of croatian breast cancer patients. Life. 2022;12:1559. 36294994 10.3390/life12101559 PMC9605052

[64] Ueng S‐H , et al. Phosphorylated mTOR expression correlates with poor outcome in early‐stage triple negative breast carcinomas. Int J Clin Exp Pathol. 2013;5:806‐813. PMC3466984 23071863

[65] Walsh S , Flanagan L , Quinn C , et al. mTOR in breast cancer: differential expression in triple‐negative and non‐triple‐negative tumors. Breast Edinb Scotl. 2012;21:178‐182. 10.1016/j.breast.2011.09.008 21963359

[66] Bryant C , Rawlinson R , Massey AJ . Chk1 Inhibition as a novel therapeutic strategy for treating triple‐negative breast and ovarian cancers. BMC Cancer. 2014;14:570. 25104095 10.1186/1471-2407-14-570 PMC4137066

[67] Zhou Z‐R , Yang Z‐Z , Wang S‐J , et al. The Chk1 inhibitor MK‐8776 increases the radiosensitivity of human triple‐negative breast cancer by inhibiting autophagy. Acta Pharmacol Sin. 2017;38:513‐523. 28042876 10.1038/aps.2016.136 PMC5386307

[68] Kai K , Kondo K , Wang X , et al. Antitumor activity of KW‐2450 against triple‐negative breast cancer by inhibiting Aurora A and B kinases. Mol Cancer Ther. 2015;14:2687‐2699. 26443806 10.1158/1535-7163.MCT-15-0096 PMC4674309

[69] Song C , Lowe VJ , Lee S . Inhibition of Cdc20 suppresses the metastasis in triple negative breast cancer (TNBC). Breast Cancer. 2021;28:1073‐1086. 33813687 10.1007/s12282-021-01242-z

[70] Romanelli A , Clark A , Assayag F , et al. Inhibiting aurora kinases reduces tumor growth and suppresses tumor recurrence after chemotherapy in patient‐derived triple‐negative breast cancer xenografts. Mol Cancer Ther. 2012;11:2693‐2703. 23012245 10.1158/1535-7163.MCT-12-0441-T

[71] Ma CX , Cai S , Li S , et al. Targeting Chk1 in p53‐deficient triple‐negative breast cancer is therapeutically beneficial in human‐in‐mouse tumor models. J Clin Invest. 2012;122:1541‐1552. 22446188 10.1172/JCI58765 PMC3314455

[72] Honma N , Horii R , Ito Y , et al. Differences in clinical importance of Bcl‐2 in breast cancer according to hormone receptors status or adjuvant endocrine therapy. BMC Cancer. 2015;15:698. 26472348 10.1186/s12885-015-1686-y PMC4607008

[73] Merino D , Lok SW , Visvader JE , Lindeman GJ . Targeting BCL‐2 to enhance vulnerability to therapy in estrogen receptor‐positive breast cancer. Oncogene. 2016;35:1877‐1887. 26257067 10.1038/onc.2015.287

[74] Terranova‐Barberio M , Thomas S , Ali N , et al. HDAC inhibition potentiates immunotherapy in triple negative breast cancer. Oncotarget. 2017;8:114156. 29371976 10.18632/oncotarget.23169 PMC5768393

[75] Maiti A , et al. Class I histone deacetylase inhibitor suppresses vasculogenic mimicry by enhancing the expression of tumor suppressor and anti‐angiogenesis genes in aggressive human TNBC cells. Int J Oncol. 2019;55:116‐130. 31059004 10.3892/ijo.2019.4796 PMC6561627

[76] Giltnane JM , Balko JM . Rationale for targeting the Ras/MAPK pathway in triple‐negative breast cancer. Discov Med. 2014;17:275‐283. 24882719

[77] Jiang W , Wang X , Zhang C , Xue L , Yang L . Expression and clinical significance of MAPK and EGFR in triple‑negative breast cancer. Oncol Lett. 2020;19:1842‐1848. 32194678 10.3892/ol.2020.11274 PMC7038935

[78] Balko JM , Schwarz LJ , Bhola NE , et al. Activation of MAPK pathways due to DUSP4 loss promotes cancer stem cell‐like phenotypes in basal‐like breast cancer. Cancer Res. 2013;73:6346‐6358. 23966295 10.1158/0008-5472.CAN-13-1385 PMC4090144

[79] Bartholomeusz C , Xie X , Pitner MK , et al. MEK inhibitor selumetinib (AZD6244; ARRY‐142886) prevents lung metastasis in a triple‐negative breast cancer xenograft model. Mol Cancer Ther. 2015;14:2773‐2781. 26384399 10.1158/1535-7163.MCT-15-0243 PMC4674314

[80] Matossian MD , Hoang VT , Burks HE , et al. Constitutive activation of MEK5 promotes a mesenchymal and migratory cell phenotype in triple negative breast cancer. Oncoscience. 2021;8:64. 34026925 10.18632/oncoscience.535 PMC8131078

[81] Coussy F , Lavigne M , De Koning L , et al. Response to mTOR and PI3K inhibitors in enzalutamide‐resistant luminal androgen receptor triple‐negative breast cancer patient‐derived xenografts. Theranostics. 2020;10:1531. 32042320 10.7150/thno.36182 PMC6993232

[82] Basho RK , Zhao Li , White JB , et al. Comprehensive analysis identifies variability in PI3K pathway alterations in triple‐negative breast cancer subtypes. JCO Precis Oncol. 2024;8:e2300124. 38484209 10.1200/PO.23.00124 PMC10954064

[83] Kumar S , Bal A , Das A , et al. Spectrum of PIK3CA/AKT mutations across molecular subtypes of triple‐negative breast cancer. Breast Cancer Res Treat. 2021;187:625‐633. 33954864 10.1007/s10549-021-06242-3

[84] Azizi E , Carr AJ , Plitas G , et al. Single‐cell map of diverse immune phenotypes in the breast tumor microenvironment. Cell. 2018;174:1293‐1308. 29961579 10.1016/j.cell.2018.05.060 PMC6348010

[85] Derose YS , Wang G , Lin Yi‐C , et al. Tumor grafts derived from women with breast cancer authentically reflect tumor pathology, growth, metastasis and disease outcomes. Nat Med. 2011;17:1514‐1520. 22019887 10.1038/nm.2454 PMC3553601

[86] Eirew P , Steif A , Khattra J , et al. Dynamics of genomic clones in breast cancer patient xenografts at single‐cell resolution. Nature. 2015;518:422‐426. 25470049 10.1038/nature13952 PMC4864027

[87] Sachs N , De Ligt J , Kopper O , et al. A living biobank of breast cancer organoids captures disease heterogeneity. Cell. 2018;172:373‐386. 29224780 10.1016/j.cell.2017.11.010

[88] Dieci MV , Criscitiello C , Goubar A , et al. Prognostic value of tumor‐infiltrating lymphocytes on residual disease after primary chemotherapy for triple‐negative breast cancer: a retrospective multicenter study. Ann Oncol. 2014;25:611‐618. 24401929 10.1093/annonc/mdt556 PMC3933248

[89] Disis ML , Stanton SE . Triple‐negative breast cancer: immune modulation as the new treatment paradigm. Am Soc Clin Oncol Educ Book. 2015:e25‐e30. doi:10.14694/EdBook_AM.2015.35.e25 25993181

[90] Denkert C , Von Minckwitz G , Darb‐Esfahani S , et al. Tumour‐infiltrating lymphocytes and prognosis in different subtypes of breast cancer: a pooled analysis of 3771 patients treated with neoadjuvant therapy. Lancet Oncol. 2018;19:40‐50. 29233559 10.1016/S1470-2045(17)30904-X

[91] Bertucci F , Finetti P , Goncalves A , Birnbaum D . The therapeutic response of ER+/HER2− breast cancers differs according to the molecular Basal or Luminal subtype. Npj Breast Cancer. 2020;6:1‐7. 32195331 10.1038/s41523-020-0151-5 PMC7060267

[92] Küng A , Strickmann DB , Galanski MS , Keppler BK . Comparison of the binding behavior of oxaliplatin, cisplatin and analogues to 5′‐GMP in the presence of sulfur‐containing molecules by means of capillary electrophoresis and electrospray mass spectrometry. J Inorg Biochem. 2001;86:691‐698. 11583787 10.1016/s0162-0134(01)00225-2

[93] Li H , Sun X , Li J , et al. Hypoxia induces docetaxel resistance in triple‐negative breast cancer via the HIF‐1α/miR‐494/Survivin signaling pathway. Neoplasia. 2022;32:100821. 35985176 10.1016/j.neo.2022.100821 PMC9403568

[94] Nedeljković M , Damjanović A . Mechanisms of chemotherapy resistance in triple‐negative breast cancer – how we can rise to the challenge. Cells. 2019;8:957. 31443516 10.3390/cells8090957 PMC6770896

[95] Krawczyk Z , Gogler‐Pigłowska A , Sojka DR , Scieglinska D . The role of heat shock proteins in cisplatin resistance. Anti‐Cancer Agents Med Chem‐ Anti‐Cancer Agents. 2018;18:2093‐2109. 10.2174/1871520618666180817114952 30156165

[96] Shen WH , Balajee AS , Wang J , et al. Essential role for nuclear PTEN in maintaining chromosomal integrity. Cell. 2007;128:157‐170. 17218262 10.1016/j.cell.2006.11.042

[97] Gupta A , Yang Q , Pandita RK , et al. Cell cycle checkpoint defects contribute to genomic instability in PTEN deficient cells independent of DNA DSB repair. Cell Cycle. 2009;8:2198‐2210. 19502790 10.4161/cc.8.14.8947

[98] Zhao J‐L , Yang J , Li Ke , et al. Abrogation of ATR function preferentially augments cisplatin‐induced cytotoxicity in PTEN‐deficient breast cancer cells. Chem Biol Interact. 2023;385:110740. 37802411 10.1016/j.cbi.2023.110740

[99] Solaini G , Sgarbi G , Baracca A . Oxidative phosphorylation in cancer cells. Biochim Biophys Acta BBA – Bioenerg. 2011;1807:534‐542. 10.1016/j.bbabio.2010.09.003 20849810

[100] Semenza GL . Defining the role of hypoxia‐inducible factor 1 in cancer biology and therapeutics. Oncogene. 2010;29:625‐634. 19946328 10.1038/onc.2009.441 PMC2969168

[101] Liao C , Glodowski CR , Fan C , et al. Integrated metabolic profiling and transcriptional analysis reveals therapeutic modalities for targeting rapidly proliferating breast cancers. Cancer Res. 2022;82:665‐680. 34911787 10.1158/0008-5472.CAN-21-2745 PMC8857046

[102] Brett JO , Spring LM , Bardia A , Wander SA . ESR1 mutation as an emerging clinical biomarker in metastatic hormone receptor‐positive breast cancer. Breast Cancer Res. 2021;23:85. 34392831 10.1186/s13058-021-01462-3 PMC8365900

[103] Hoy SM . Elacestrant: first approval. Drugs. 2023;83:555‐561. 37060385 10.1007/s40265-023-01861-0 PMC10667141

[104] Stella S , Martorana F , Massimino M , Vitale SR , Manzella L , Vigneri P . Potential therapeutic targets for luminal androgen receptor breast cancer: what we know so far. OncoTargets Ther. 2023;16:235‐247. 10.2147/OTT.S379867 PMC10089148 37056632

[105] Gerratana L , Basile D , Buono G , et al. Androgen receptor in triple negative breast cancer: a potential target for the targetless subtype. Cancer Treat Rev;68:2018. 10.1016/j.ctrv.2018.06.005 29940524

[106] Hicks SC , Townes FW , Teng M , Irizarry RA . Missing data and technical variability in single‐cell RNA‐sequencing experiments. Biostatistics. 2018;19:562‐578. 29121214 10.1093/biostatistics/kxx053 PMC6215955

[107] Rashid NS , Hairr NS , Murray G , et al. Identification of nuclear export inhibitor‐based combination therapies in preclinical models of triple‐negative breast cancer. Transl Oncol. 2021;14:10123. 10.1016/j.tranon.2021.101235 PMC8512760 34628286

[108] Turner TH , Alzubi MA , Sohal SS , Olex AL , Dozmorov MG , Harrell JC . Characterizing the efficacy of cancer therapeutics in patient‐derived xenograft models of metastatic breast cancer. Breast Cancer Res Treat. 2018;170:221‐234. 29532339 10.1007/s10549-018-4748-4

[109] Miller TE , Mack SC , Rich JN . Mouse Cell Depletion. Miltenyi Biotec.

[110] Chen Y , Pal B , Lindeman GJ , Visvader JE , Smyth GK . R code and downstream analysis objects for the scRNA‐seq atlas of normal and tumorigenic human breast tissue. Sci Data. 2022;9:96. 35322042 10.1038/s41597-022-01236-2 PMC8943201

[111] Andrews S . FastQC: a quality control tool for high throughput sequence data. 2018. Accessed October 9, 2024. Available online: https://www.bioinformatics.babraham.ac.uk/projects/fastqc/

[112] Ewels P , Magnusson M , Lundin S , Käller M . MultiQC: summarize analysis results for multiple tools and samples in a single report. Bioinformatics. 2016;32(19):3047–3048. doi:10.1093/bioinformatics/btw354 27312411 PMC5039924

[113] Cell ranger secondary analysis outputs—official 10x Genomics Support. 10x Genomics. Accessed February 27, 2024. https://www.10xgenomics.com/support/software/cell‐ranger/latest/analysis/outputs/cr‐outputs‐secondary‐analysis

[114] Hao Y , Hao S , Andersen‐Nissen E , et al. Integrated analysis of multimodal single‐cell data. Cell. 2021;184:3573‐3587. 34062119 10.1016/j.cell.2021.04.048 PMC8238499

[115] Common Considerations for Quality Control Filters for Single Cell RNA‐seq Data. 10x Genomics. Accessed September 4, 2024. https://www.10xgenomics.com/analysis‐guides/common‐considerations‐for‐quality‐control‐filters‐for‐single‐cell‐rna‐seq‐data

[116] Ahlmann‐Eltze C , Huber W . Comparison of transformations for single‐cell RNA‐seq data. Nat Methods. 2023;20:665‐672. 37037999 10.1038/s41592-023-01814-1 PMC10172138

[117] Dave A , Charytonowicz D , Francoeur NJ , et al. The Breast Cancer Single‐Cell Atlas: defining cellular heterogeneity within model cell lines and primary tumors to inform disease subtype, stemness, and treatment options. Cell Oncol. 2023;46:603‐628. 10.1007/s13402-022-00765-7 PMC10205851 36598637

[118] Antonsson SE , Melsted P . Batch correction methods used in single cell RNA‐sequencing analyses are often poorly calibrated. bioRxiv. 2024;2024.03.19.585562 Preprint at doi:10.1101/2024.03.19585562 PMC12315870 40623818

[119] Krämer A , Green J , Pollard J , Tugendreich S . Causal analysis approaches in Ingenuity Pathway Analysis. Bioinforma Oxf Engl. 2014;30:523‐530. 10.1093/bioinformatics/btt703 PMC3928520 24336805

[120] Choi J‐H , In Kim H , Woo HG . scTyper: a comprehensive pipeline for the cell typing analysis of single‐cell RNA‐seq data. BMC Bioinformatics. 2020;21:342. doi:10.1186/s12859-020-03700-5 32753029 PMC7430822

[121] Tickle T , Tirosh I , Georgescu C , Brown M , Haas B . inferCNV of the Trinity CTAT Project. Cambridge, MA, USA; Klarman Cell Observatory, Broad Institute of MIT and Harvard; 2019. https://github.com/broadinstitute/inferCNV

[122] omicsCore/scTyper . omicsCore 2024.

[123] WCCTR_RNASeq_Pipeline/SingleCell/cells2keep_convertMerged2Unmerged_050823.R at master · AmyOlex/WCCTR_RNASeq_Pipeline. GitHub. Accessed February 27, 2024. https://github.com/AmyOlex/WCCTR_RNASeq_Pipeline/blob/master/SingleCell/cells2keep_convertMerged2Unmerged_050823.R

[124] Love MI , Huber W , Anders S . Moderated estimation of fold change and dispersion for RNA‐seq data with DESeq2. Genome Biol. 2014;15:550. 25516281 10.1186/s13059-014-0550-8 PMC4302049

[125] BrCa_cell_atlas/scSubtype at main Swarbricklab‐code/BrCa_cell_atlas. GitHub. Accessed February 27, 2024. https://github.com/Swarbricklab‐code/BrCa_cell_atlas/tree/main/scSubtype

[126] Chen Xi , Li J , Gray WH , et al. TNBCtype: a subtyping tool for triple‐negative breast cancer. Cancer Inform. 2012;11:147‐156. 22872785 10.4137/CIN.S9983 PMC3412597

