# Supplementary figures and images for: Single‐cell transcriptional atlas of human breast cancers and model systems
Source: Clin Transl Med. 2024 Oct 17;14(10):e70044. doi: 10.1002/ctm2.70044 (PMC11483560; doi:10.1002/ctm2.70044)

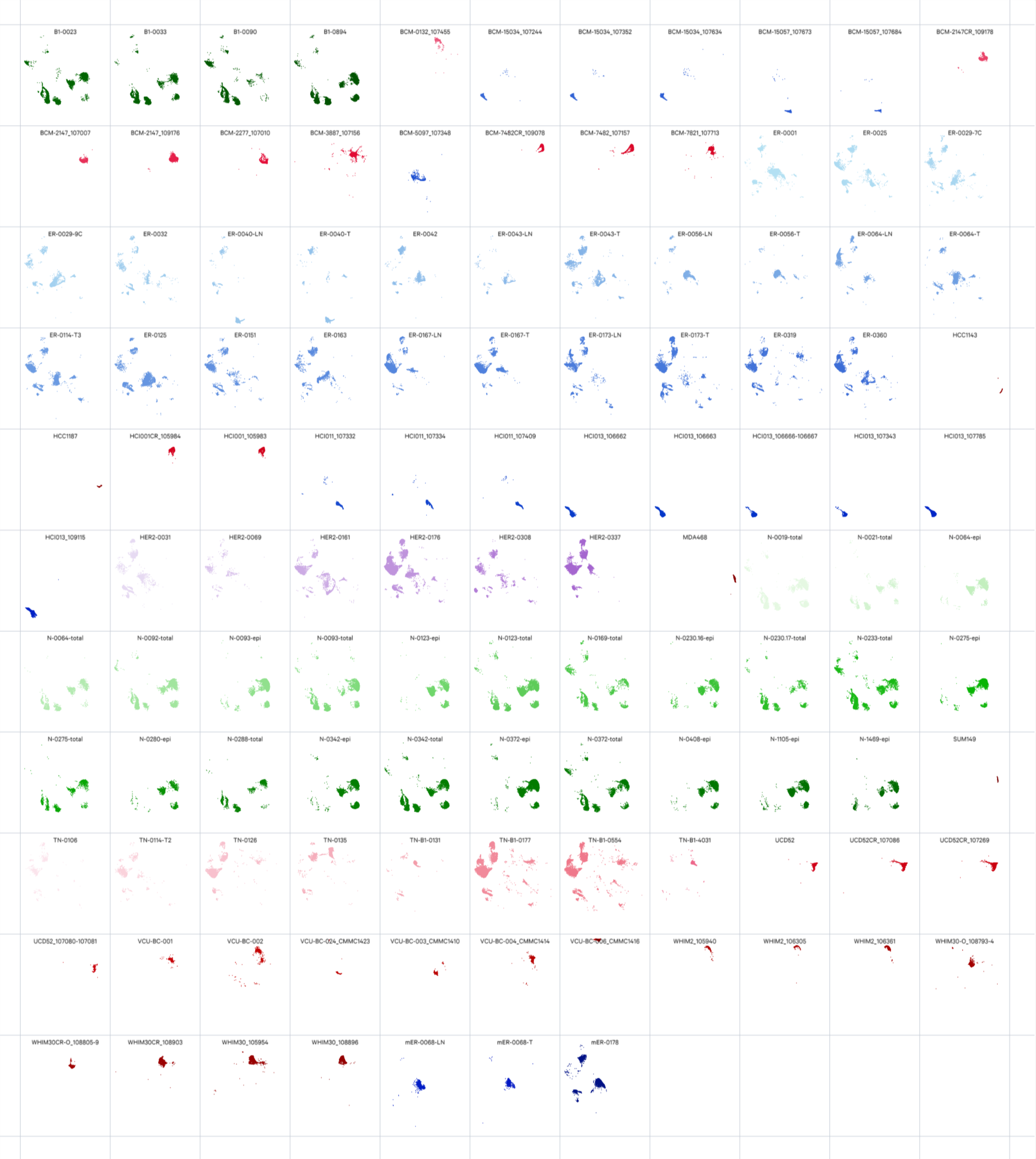

Supplement: Supplementary file 1 — Supporting information [file CTM2-14-e70044-s003.jpeg]

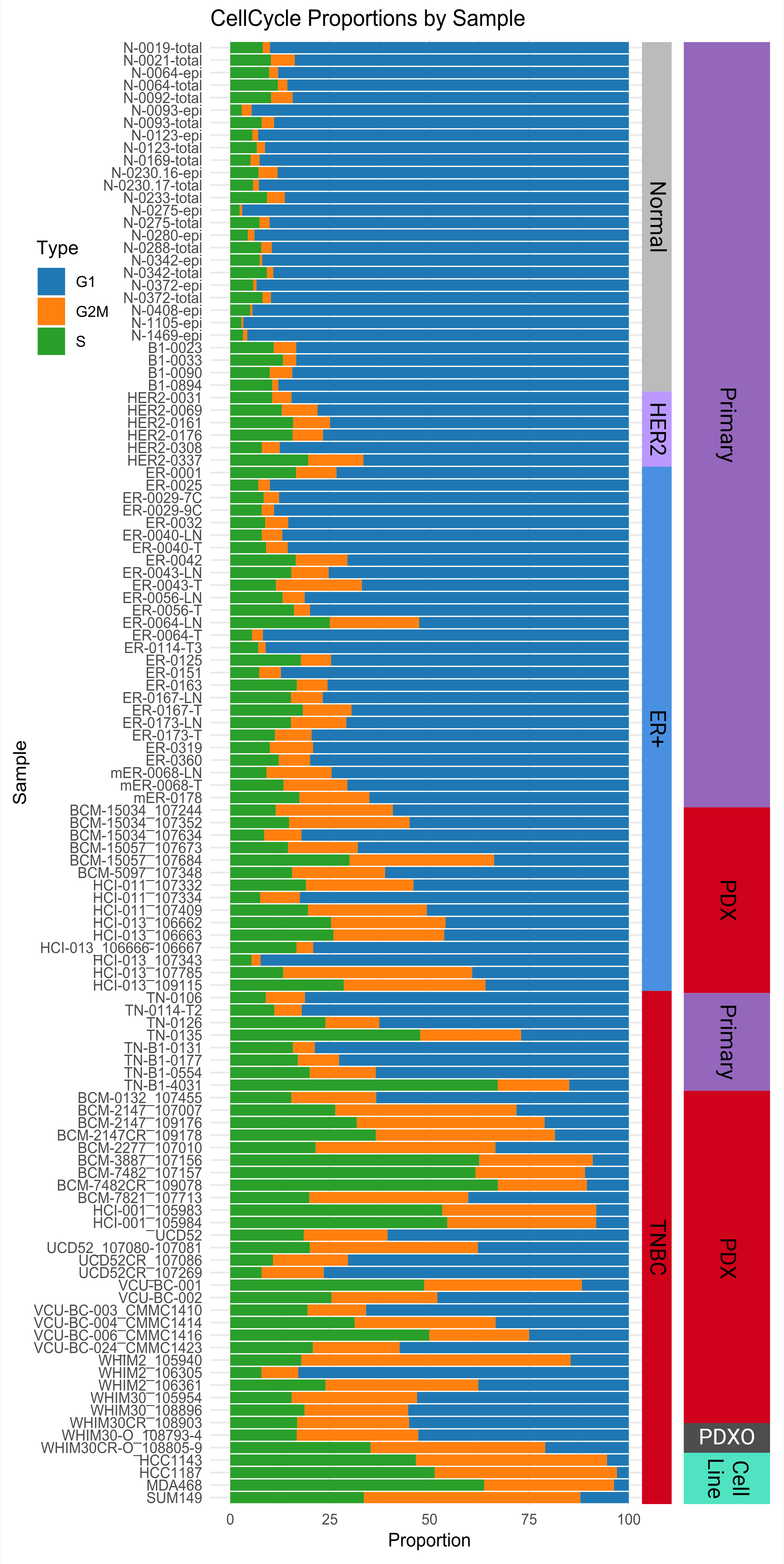

Supplement: Supplementary file 2 — Supporting information [file CTM2-14-e70044-s004.jpeg]

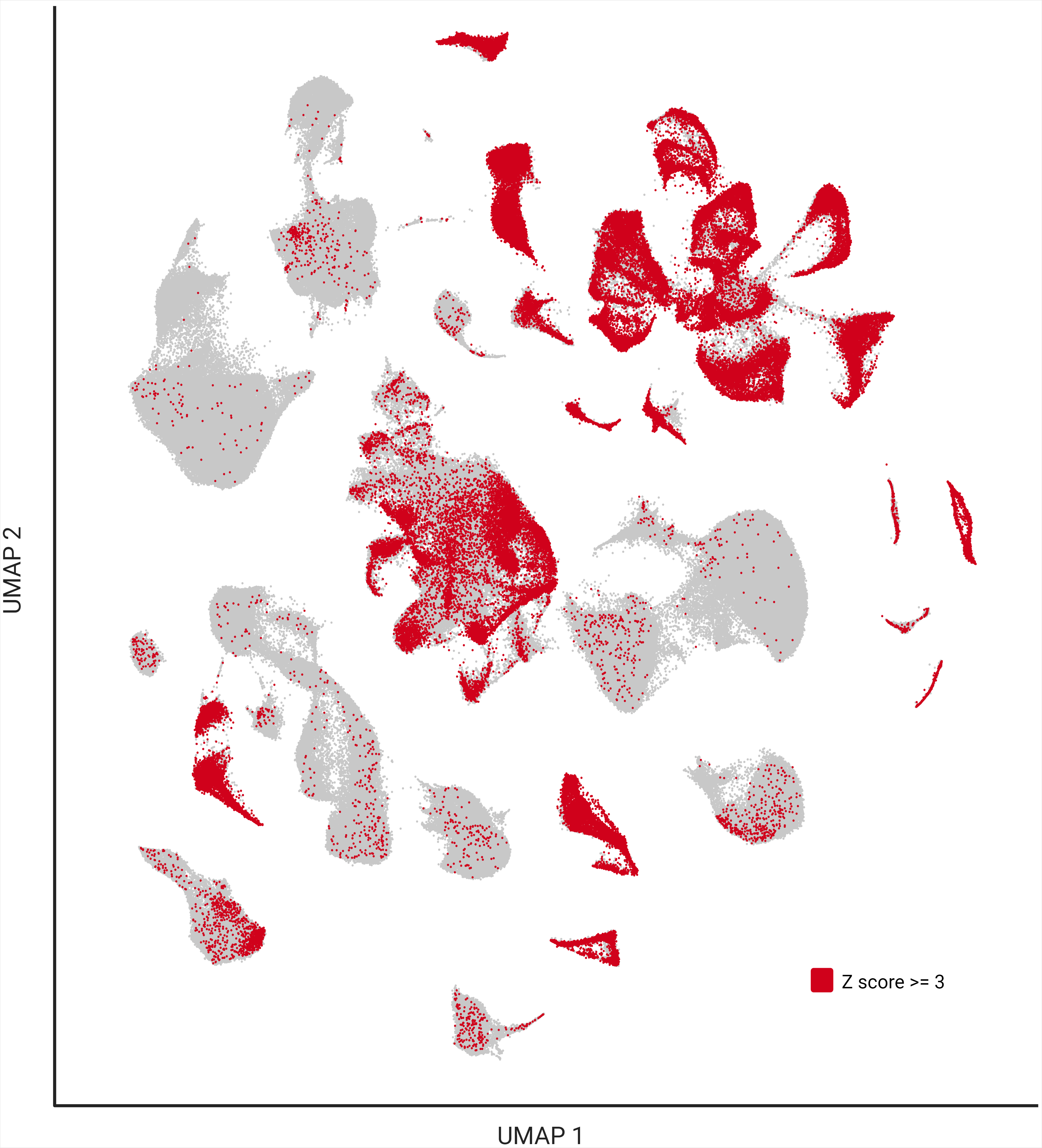

Supplement: Supplementary file 3 — Supporting information [file CTM2-14-e70044-s002.jpeg]
